# Caregiver perspectives enable accurate diagnosis of neurodegenerative disease

**DOI:** 10.1002/alz.14377

**Published:** 2024-11-19

**Authors:** Alexander G. Murley, Lucy Bowns, Marta Camacho, Caroline H. Williams‐Gray, Kamen A. Tsvetanov, Timothy Rittman, Roger A. Barker, John T. O'Brien, James B. Rowe

**Affiliations:** ^1^ Department of Clinical Neurosciences University of Cambridge Cambridge UK; ^2^ Cambridge University Hospitals NHS Foundation Trust Cambridge UK; ^3^ Department of Psychology University of Cambridge Cambridge UK; ^4^ Department of Psychiatry University of Cambridge Cambridge UK; ^5^ MRC Cognition and Brain Sciences Unit University of Cambridge Cambridge UK

**Keywords:** behavior, caregiver, dementia, diagnosis, informant, neurodegeneration, relative

## Abstract

**BACKGROUND:**

The history from a relative or caregiver is an important tool for differentiating neurodegenerative disease. We characterized patterns of caregiver questionnaire responses, at diagnosis and follow‐up, on the Cambridge Behavioural Inventory (CBI).

**METHODS:**

Data‐driven multivariate analysis (*n* = 4952 questionnaires) was undertaken for participants (*n* = 2481) with Alzheimer's disease (typical/amnestic *n* = 543, language *n* = 50, and posterior cortical *n* = 50 presentations), Parkinson's disease (*n* = 740), dementia with Lewy bodies (*n* = 55), multiple system atrophy (*n* = 55), progressive supranuclear palsy (*n* = 422), corticobasal syndrome (*n* = 176), behavioral variant frontotemporal dementia (*n* = 218), semantic (*n* = 125) and non‐fluent variant progressive aphasia (*n* = 88), and motor neuron disease (*n* = 12).

**RESULTS:**

Item‐level support vector machine learning gave high diagnostic accuracy between diseases (area under the curve mean 0.83), despite transdiagnostic changes in memory, behavior, and everyday function. There was progression in CBI subscores over time, which varied by diagnosis.

**DISCUSSION:**

Our results highlight the differential diagnostic information for a wide range of neurodegenerative diseases contained in a simple, structured collateral history.

**Highlights:**

- We analyzed 4952 questionnaires from caregivers of 2481 participants with neurodegenerative disease.
- Behavioral and neuropsychiatric manifestations of neurodegenerative disease had overlapping diagnostic boundaries.
- Simple questionnaire response patterns were sufficient for accurate diagnosis of each disease.
- We reinforce the value of a collateral history to support a diagnosis of dementia.
- The Cambridge Behavioural Inventory is sensitive to change over time and suitable as an outcome measure in clinical trials.

## 1 BACKGROUND

Accurate diagnosis is an essential step for the clinical care, management, and research of patients with neurodegenerative disorders. An ideal diagnostic test or tool would be cheap, safe, scalable, and accurate across multiple diseases. Commonly used technological approaches, based on magnetic resonance imaging,^1^, ^2^ positron emission tomography,^3^, ^4^, ^5^ plasma,^5^, ^6^, ^7^ and cerebrospinal fluid^6^, ^8^, ^9^, ^10^ meet some but not all of these criteria, while novel digital technologies and wearable devices^11^, ^12^ are still in development. Here we consider an alternative approach, based on the detailed analysis of caregiver‐reported behavioral and functional changes that frequently accompany neurodegenerative diseases.

A diagnosis of dementia is based not just on an affected person's symptoms or manifest signs of disease, but also often the account of a caregiver, relative, or other informant.^13^ This perspective from a third party (the informant) may differ from that of the person with the disease, especially when the illness affects insight into the effects of the illness, and may convey disease‐specific information. A collateral history is recommended for clinical diagnosis of dementias, and is useful for care planning.^13^ Clinical trials may also require an informant contribution to clinically meaningful endpoints.^14^

Formalizing informant collateral information can be achieved with structured questionnaires. For example, many studies of dementia have used the Neuropsychiatric Inventory (NPI), which is completed by a clinician after a caregiver interview.^15^, ^16^, ^17^ Other scales measure the impact of dementia on a caregiver's quality of life.^18^ The Cambridge Behavioural Inventory (CBI), revised in 2008 to a short version (CBI‐R), is completed by a caregiver without professional input.^19^, ^20^ They are asked to rate the frequency of 45 behaviors over the preceding month. Question domains relate to memory and orientation, everyday skills, self‐care, abnormal behavior, mood, abnormal beliefs, eating habits, sleep, stereotyped motor behaviors, and motivation. The CBI‐R correlates well with other ratings that are completed by the clinician or researcher.^15^ CBI‐R total and subsection scores statistically differ between neurodegenerative diseases.^20^ The CBI‐R is also sensitive to prodromal behavioral impairment in genetic frontotemporal dementia (FTD).^21^ However, such group‐wise differences are not the same as differential diagnostic accuracy.

We proposed that item‐level analyses would improve the diagnostic differentiation between disorders. To test this, we used the number and distribution of endorsements over the CBI‐R questions, from informants regarding people with 1 of 12 clinical disorders: Alzheimer's disease (AD; including the typical/amnestic, language, and posterior cortical clinical presentations); Parkinson's disease (PD); dementia with Lewy bodies (DLB); multiple system atrophy (MSA); and the syndromes associated with frontotemporal lobar degeneration including behavioral variant frontotemporal dementia (bvFTD), the semantic and non‐fluent variants of primary progressive aphasia (svPPA and nfvPPA, respectively), progressive supranuclear palsy (PSP), and corticobasal syndrome (CBS). The CBI and CBI‐R were delivered in paper format, by post, but are also suitable for administration digitally by tablet or secure web‐browser. They are a priori cheap, safe, and scalable so the critical assessment is the accuracy across multiple different diseases.

Our study had two principal aims. First, we aimed to test the sensitivity of informant observations of fine‐grained behavioral features: Could item‐level analysis support accurate individual differentiation of patients drawn from each of 12 diagnostic groups? Second, we wanted to provide support for transdiagnostic trials of symptomatic therapies in neurodegenerative disease: To what extent are some behavioral profiles similar across diagnostic groups? A transdiagnostic approach, which investigates clinical features and their neurobiological basis across multiple diagnoses, has been suggested for psychiatric^22^, ^23^ and neurological^24^, ^25^ diseases that share clinical, pathophysiological, or functional anatomical features. While the neurodegenerative diseases above are associated with distinct molecular etiologies, they are only partially differentiated by coarse‐grained clinical domains (e.g., amnesia or apathy). Clinical trials testing interventions for behavioral impairment in dementia are urgently required. Quantifying the heterogeneity and progression of symptoms, for example in CBI‐R item responses, would inform participant screening, trial design, endpoint selection, and power calculations. A subsidiary aim was therefore to test the hypothesis that trials’ inclusion based on symptom profile, rather than a specific clinical diagnosis, would have greater power to detect a meaningful reduction in symptoms.

## 2 METHODS

### 2.1 Participant recruitment and assessment

Participants attended either the memory disorders or movement disorders clinics at Cambridge University Hospitals National Health Service (NHS) Trust (known also as Addenbrookes Hospital) or the Parkinson's Disease Research Clinic at the John van Geest Centre for Brain Repair, University of Cambridge. Participants provided written informed consent or, if they lacked capacity to consent, their next of kin was consulted as established in UK law. During this consultee process, the next of kin of a potential participant advised the researcher on what the participant's wishes and feelings on participating in the study would have been if they were able to consent for themselves.^26^ All data were fully anonymized before analysis. Data were collected using paper questionnaires then stored using a REDCap electronic data capture system hosted at the University of Cambridge (Cambridge, UK).^27^ The 81‐item CBI was used until 2008 and a subset of questions was retained in the revised 45‐item version (CBI‐R) used thereafter. Contemporary cognitive assessment using the revised Addenbrooke's Cognitive Examination (ACE‐R)^28^ was performed at the clinical (NHS healthcare) assessment along with a comprehensive clinical assessment, diagnostic neuropsychology, neuroimaging, and other investigations as indicated for diagnosis. For participants attending the Parkinson's Disease Research Clinic, contemporary assessment included confirmation of diagnosis (UK Brain Bank criteria for PD); collection of demographic, comorbidity, and medication data; the Unified Parkinson's Disease Rating Scale (UPDRS) or Movement Disorders Society Modified UPDRS; and neuropsychological assessment including the ACE‐R.

We included all patients with a clinical diagnosis belonging to one of the three common neurodegenerative disease groups: (1) AD, including typical amnestic or multi‐domain disease,^29^, ^30^ posterior cortical atrophy (PCA),^31^, ^32^ and logopenic variant or mixed primary progressive aphasia (PPA)^33^ subtypes; (2) alpha‐synucleinopathies including DLB,^34^, ^35^ PD,^36^, ^37^ and MSA;^38^ and (3) clinical syndromes associated with frontotemporal lobar degeneration including bvFTD,^39^, ^40^ nfvPPA,^33^ svPPA,^33^, ^41^ PSP,^42^, ^43^ and CBS.^44^ We grouped logopenic variant and mixed PPA into a language variant of a probable AD (lvAD) group, as patients with these phenotypes usually have AD pathology *post mortem*.^45^, ^46^ A small number of patients seen in the above clinics with motor neuron disease (MND), and not dementia during follow‐up, were included. We did not include participants with prodromal symptoms (e.g., mild cognitive impairment) or non‐degenerative causes of dementia (e.g., vascular dementia), to benefit from higher clinico‐pathological correlations.

All participants were assessed by a senior clinician with subspeciality expertise in dementia or movement disorders, supported by a multidisciplinary team review meeting. The clinical diagnosis was made using contemporaneous consensus diagnostic criteria. The clinical diagnosis reported here is the final clinical diagnosis at last patient contact. For example, an initial diagnosis of CBS revised at follow‐up to PSP would be labelled in this study as PSP. We did not retrospectively change the clinical diagnosis for the subset of patients with *post mortem* neuropathology. For example, a patient with a final clinical diagnosis of CBS with a neuropathological diagnosis of AD would remain labelled in this study as CBS. The CBI‐R questionnaire result was typically available to the clinician at the diagnostic visit, but a diagnosis was not made based on the CBI‐R alone but with reference to history from the patient, cognitive and neurological examination, informant history, and ancillary investigations (e.g., imaging, blood tests, and in some cases other biomarkers) after discussion within a multidisciplinary team after each clinic.

**RESEARCH IN CONTEXT**

1. **Systematic review**: We reviewed the literature using traditional sources. Caregiver perspectives convey clinically meaningful information, distinct from that disclosed by a participant or elicited by a clinician. There are many validated questionnaires for caregivers and relatives of participants with neurodegenerative disease; however, they are typically tested on relatively small cohorts with a limited range of diagnoses.
2. **Interpretation**: Our findings in a large cohort of participants reveal overlapping symptom profiles across 12 different neurodegenerative diseases. Patterns of item‐level responses provided high diagnostic accuracy between diseases. Longitudinal progression in scores varied by diagnosis and questionnaire subscore.
3. **Future directions**: Our results reinforce the diagnostic value of collateral information. They provide a benchmark against which to evaluate more complex, invasive, expensive, or technology‐based diagnostic tools. Caregiver perspectives, as measured by the Cambridge Behavioural Inventory, may be a meaningful endpoint in clinical trials.

Clinical diagnosis was the best available standard to test caregiver perspectives against on the whole cohort. Neuropathological diagnosis was only available in a small subset of cases. We chose final diagnosis as likely the most accurate in vivo clinical diagnosis. Diagnostic accuracy increases over time in neurodegenerative disease, as individuals accumulate clinical features with progression that are more likely to match a canonical diagnostic phenotype, and as further diagnostic tests or response to treatment become available to clinicians.

The first available CBI‐R was used in all individuals. In all disease groups except PD this was the first visit to a NHS memory clinic at our center. Data for most participants with PD came from the Parkinson's Disease Research Clinic, which recruits participants within 2 years from diagnosis.

Longitudinal assessments were available for a subset of participants (total *n* = 1037, AD = 173, lv/other PPA = 24, PCA = 25, DLB = 56, PD = 150, bvFTD = 133, PSP = 199, CBS = 95, MSA = 24, nfvPPA = 61, svPPA = 95, MND = 2) over a mean follow‐up time of 2.7 years (standard deviation [SD] = 2.6) and mean 2.7 visits (SD = 2.2).

### 2.2 Statistical analysis

For data collected before 2008 the 45 items included in the CBI‐R were extracted from the 81 CBI responses. We classified informants as follows: husband/unmarried male partners, wife/unmarried female partners, son, daughter, other friend/family and health‐care professional. The health‐care professional group included staff who knew the participant prior to the appointment (for example care home staff) and were not memory clinic staff.

We excluded questionnaires if > 10% of the questions were unanswered (*n* = 357, 6.6%) or if all questions in one of the subsections were missing (*n* = 373, 6.9%). For the summary statistics we used the total score as a proportion of all answered questions, as recommended by the CBI‐R authors.^19^ For principal component analysis and support vector machine learning we replaced missing data with the mean score for the respective CBI‐R subsection.

We used principal component analysis to reduce the dimensionality of the responses, and identify components of covarying questions across the cohort. This enabled us to track the multidimensional impairments across diseases at a subject level, in terms of individual loadings onto each component, to reveal the pattern of deficits observed by informants. Principal component analysis was performed on the baseline CBI‐R questionnaires only. A Kaiser–Meyer–Olkin (KMO) test confirmed our dataset was suitable for principal component analysis (KMO = 0.96).

We selected components using permutation‐based bootstrap principal component analysis, followed by varimax rotation. This method is described in detail elsewhere.^47^, ^48^, ^49^, ^50^ Bootstrap sampling involved 1000 bootstrap samples generated from the original dataset. Principal component analysis was then performed on each of these bootstrap samples to calculate the explained variance (median across 1000 bootstraps) of the principal components. Next, the null distribution for each component was generated. For each variable in the original dataset values were randomly shuffled (permuted) before the bootstrap principal component analysis. This was repeated 1000 times to create a null distribution of explained variance for each component with the null hypothesis that there is no meaningful underlying structure in the data. Finally, for significance testing the explained variance of each component from the principal component analysis on original, unshuffled data was compared to the corresponding null distribution created in the previous step. We selected seven components with explained variance significantly (*p* < 0.05) higher than that of the corresponding distribution from the permuted data.

We tested the classification accuracy of the CBI‐R using supervised machine (SVM) learning, specifically SVM learning with a linear kernel (see Pisner and Schnyer^47^ and Myszczynska et al.^48^ for reviews of SVM methods). Weighting was used to correct for unbalanced groups. We trained and tested two types of SVM on the baseline CBI‐R questions. First, a “one versus all” design to determine diagnostic accuracy and receiver operating characteristic curve for each diagnosis versus all other participants. Second, a pairwise “one versus one” SVM to determine pairwise accuracy for all diagnostic pairs (e.g., AD vs. DLB). For each SVM, we separated training and testing data using 10‐fold cross‐validation to determine classification accuracy. The accuracy was determined from testing the trained model on the unseen 10% of data. This was repeated 1000 times with permuted training/test splits. Cross‐validated sequential feature elimination was used to determine the subset of features required for each one versus one SVM.

We estimated the annualized rate of change of the CBI‐R using linear mixed effects models with baseline score and follow‐up time as fixed effects and subject as a random effect.

Finally, we performed power calculations to estimate sample sizes for clinical trials for behavioral impairments in dementia. We calculated a CBI‐Behavior subscore from all questions loading on the behavioral components derived from the principal component analysis. A power calculation was performed for the three major dementias: AD‐related syndromes, including the amnestic, aphasic, and posterior cortical presentations; syndromes associated with frontotemporal lobar degeneration, including bvFTD, svPPA, nfvPPA, PSP, and CBS; and finally an alpha‐synucleinopathy group that included DLB, PD, and MSA. We also calculated the sample size required if recruitment was restricted to the subset of patients with clinically significant behavioral impairments. This was defined as > 50% of behavioral symptoms present weekly or more frequently, which translates to a CBI‐Behavior subscore of ≥ 14. We used estimates from linear mixed effects models (one model for each group) to estimate the CBI‐Behavior score 6 months from baseline (the null hypothesis mean). The alternative hypothesis (the target effect size) was a 30% reduction in CBI‐Behavior score. We calculated the sample size for a two‐sample *t* test with significance level set to 0.05 and a power of 95% using the sampsizepwr function in MATLAB. Analysis was performed in MATLAB 2022b (Mathworks) with further statistical analysis in JASP (version 0.13).

### 2.3 Data availability

Anonymized data are available on reasonable request, although restrictions may apply to comply with participant consent and anonymity constraints.

## 3 RESULTS

Four thousand nine hundred fifty‐two CBI/CBI‐R questionnaires were available. CBI/CBI‐R questionnaires were completed for 2481 participants at baseline. Diagnosis and demographic details are in Table 1. Total CBI‐R scores significantly differed between diagnoses (*F*[10] = 137.1, *p* < 0.001). Three hundred nine participants had a neuropathological diagnosis after brain donation and clinicopathological correlations are shown in Supplementary Materials S1 in supporting information. A breakdown of individual question responses, separated by group, is in Supplementary Materials S2 in supporting information.

**TABLE 1:** Patient participant demographics at baseline CBI‐R assessment.

	AD	PCA	lvAD	DLB	PD	MSA	PSP	CBS	bvFTD	nfvPPA	svPPA	MND
*N* % of total	453 18.3	50 2.0	48 1.9	94 3.8	740 29.8	55 2.2	422 17.0	176 7.1	218 8.8	88 3.5	125 5.0	12 0.5
Age mean (SD)	67.9 (9.7)	59.7 (12.1)	71.1 (5.5)	71.2 (6.8)	66.0 (9.3)	64.5 (8.2)	70.2 (7.0)	69.1 (8.2)	62.3 (9.2)	69.7 (9.2)	64.3 (7.1)	60.4 (16.1)
Sex (%M)	54.7	46.0	37.5	81.9	63.9	58.2	52.8	46.5	61.0	53.4	59.2	50.0
ACE‐R mean (SD)	63.5 (17.7)	66.0 (16.3)	55.8 (19.8)	70.9 (13.4)	91.5 (11)	81.9 (17.3)	77.4 (13.5)	71.0 (20.6)	66.6 (19.8)	66.0 (23.7)	54.3 (17.7)	79.0 (13.8)
CBI‐R mean (SD)	47.3 (32.1)	59.7 (12.1)	31.4 (29.4)	52.3 (31.4)	20.1 (19.4)	37.8 (24.3)	51.4 (31.5)	42.0 (30.1)	70.8 (32.0)	28.5 (25.8)	44.4 (26.8)	35.2 (34.0)
Disease duration at time of CBI (months)	−9.0	−2.4	−0.70	−3.31	37.6	−5.53	6.3	−4.64	−6.0	−2.27	−1.81	0.63

*Note*: Values for age, ACE‐R, and CBI‐R are mean and (SD). Age is age at baseline CBI‐R test. Sex is % male. ACE‐R scores were only available for a subset of patients (AD = 326, PCA = 40, lvAD = 44, DLB = 73, PD = 399, MSA = 33, PSP = 258, CBS = 133, bvFTD = 155, nfvPPA = 65, svPPA = 75, MND = 8). Disease duration is negative where the CBI was completed before the diagnostic appointment (e.g., if a diagnosis was only made at a later visit).

Abbreviations: ACE‐R, Addenbrooke's Cognitive Examination; AD, Alzheimer's disease; bvFTD, behavioral variant frontotemporal dementia; CBI‐R, Cambridge Behavioural Inventory short version; CBS, corticobasal syndrome; DLB, dementia with Lewy bodies; lvAD, language variants associated with Alzheimer's disease include logopenic and mixed variants of primary progressive aphasia; MND, motor neuron disease; MSA, multiple system atrophy; nfvPPA, non‐fluent variant primary progressive aphasia; PCA, posterior cortical atrophy; PD, Parkinson's disease; PSP, progressive supranuclear palsy; SD, standard deviation; svPPA, semantic variant primary progressive atrophy.

The relationship between the subject of the questionnaire and informant was available for the majority of questionnaires (*n* = 2022). We compared the CBI‐R total scores given by male (*n* = 588) and female (*n* = 1030) partners, sons (*n* = 55), daughters (*n* = 167), other family/friends (*n* = 141), and health‐care professionals (*n* = 41). Diagnosis, ACE‐R, and patient age were included as covariates. CBI‐R scores differed by the relationship between the informant and patient (*F*[5] = 12.64, *p* ≤ 0.001). Post hoc testing revealed that sons and daughters gave higher CBI‐R ratings than either male or female partners (sons: vs. male *t* = 4.8, *p* < 0.001 or female *t* = 3.46, *p* = 0.007 partners; daughters: vs. male *t* = 6.72, *p* < 0.001 or female partners *t* = 4.66, *p* < 0.001).

**FIGURE 1:**
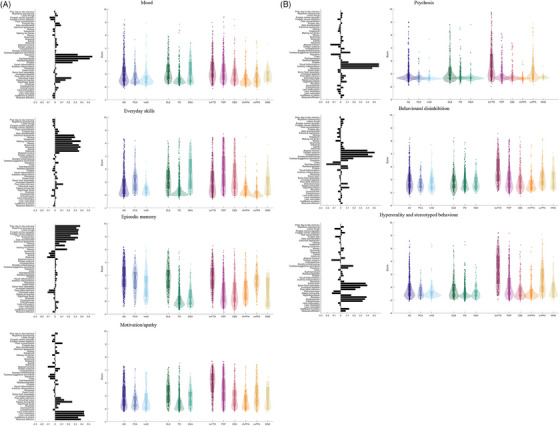
Principal component analysis of CBI‐R scores. Each row represents one of the seven components extracted from the principal component analysis (components 1–4 in plot A, 5–7 in plot B). The bar plot on the left side shows the score from each CBI‐R question on the component. The colored violin scatter boxplots show the individual loaded, grouped by disease, onto the component. AD, Alzheimer's disease; bvFTD, behavioral variant frontotemporal dementia; CBI‐R, Cambridge Behavioural Inventory short version; CBS, corticobasal syndrome; DLB, dementia with Lewy bodies; lvAD, language variants associated with Alzheimer's disease include logopenic and mixed variants of primary progressive aphasia; MND, motor neuron disease; MSA, multiple system atrophy; nfvPPA, non‐fluent variant primary progressive aphasia; PCA, posterior cortical atrophy; PD, Parkinson's disease; PSP, progressive supranuclear palsy; svPPA, semantic variant primary progressive atrophy.

Seven principal components were identified using permutation testing (Figure 1A and B). Each component represented a group of co‐varying responses from the CBI‐R. The first component reflected caregiver ratings of mood disturbance including endorsements of sad or depressed mood, crying, restlessness, agitation, and irritability. A proportion of participants with all diagnoses had high scores on this component. Principal component two represented impaired daily function, characterized by difficulty using electrical appliances, writing, using the telephone, making hot drinks, using money, eating, and getting washed and dressed. Again, a proportion of all groups had high scores, but participants with MSA, PSP, and CBS tended to have higher loadings compared to other groups at first presentation. The third component summarized caregiver or relative endorsement of impaired episodic memory, including poor day‐to‐day memory, repetitive questioning, and forgetting the names of people or places. This was expressed strongly (not surprisingly) by participants with AD (including amnestic, posterior cortical, and language presentations) and DLB, but also bvFTD. Principal component four highlighted apathetic behavior, with reduced enthusiasm, motivation, affection, and indifference. Apathy was seen across all diagnoses, but was most marked in bvFTD, PSP, DLB, and a proportion of AD participants. Informant reports of psychosis, including delusions and hallucinations, were included in component five and seen not only in DLB but also a subset of participants with PD, AD, bvFTD, and svPPA. The final two components reflected challenging behaviors, one weighted toward disinhibition and the other hyperorality and stereotyped behavior. Behavioral disinhibition was seen across all diagnostic groups but was most strongly expressed in the bvFTD and svPPA cohorts.

**FIGURE 2:**
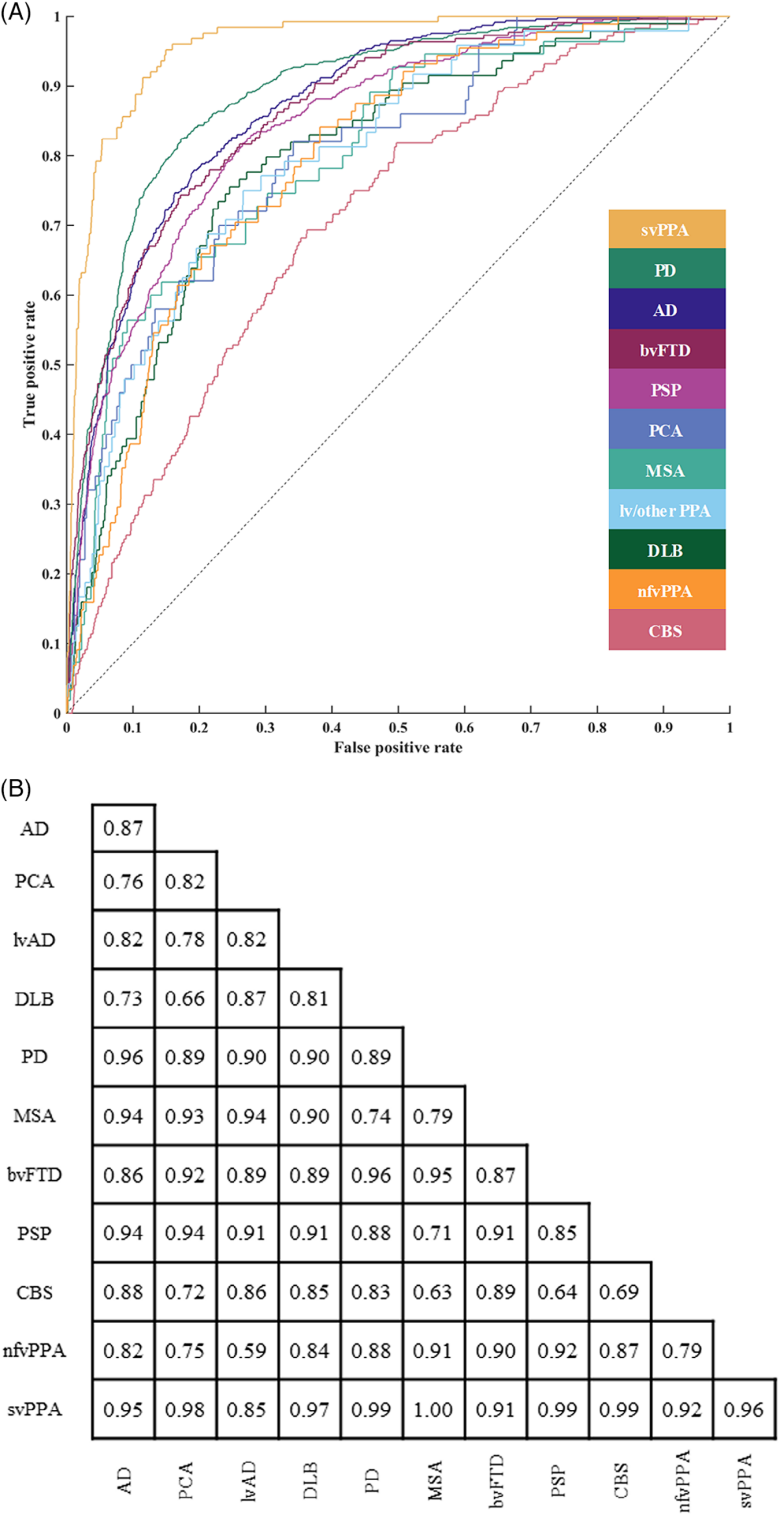
Classification accuracy of the CBI‐R. A, ROC curves for each diagnosis against all other diagnoses from a one versus all SVM. B, Area under the ROC curve (AUC) scores for all pairwise comparisons (from one vs. one SVM). The diagonal values represent the AUC scores for the one versus all SVM. AD, Alzheimer's disease; bvFTD, behavioral variant frontotemporal dementia; CBI‐R, Cambridge Behavioural Inventory short version; CBS, corticobasal syndrome; DLB, dementia with Lewy bodies; lvAD, language variants associated with Alzheimer's disease include logopenic and mixed variants of primary progressive aphasia; MND, motor neuron disease; MSA, multiple system atrophy; nfvPPA, non‐fluent variant primary progressive aphasia; PCA, posterior cortical atrophy; PD, Parkinson's disease; PSP, progressive supranuclear palsy; ROC, receiver operating characteristic; SVM, support vector machine; svPPA, semantic variant primary progressive atrophy.

Next, we tested the diagnostic utility of the CBI‐R using cross‐validated SVM learning. Participants with MND were not included in this analysis because of low numbers (*n* = 12). We tested the overall accuracy of each diagnosis against all others in a single SVM (Figure 2A). Informant responses could accurately distinguish participants with svPPA (area under the curve [AUC]: 0.96), PD (AUC: 0.89), AD (AUC: 0.87), and bvFTD (AUC: 0.87). CBS had the lowest accuracy (AUC: 0.69). We then tested the pairwise accuracy with multiple SVMs (Figure 2B). The CBI‐R also had high diagnostic accuracy in discriminating svPPA, bvFTD, PSP, PD, and MSA from most other diagnostic groups. Accuracy was lower in DLB, CBS, nfvPPA, and rarer presentations of AD (PCA and lvAD). Diagnostic accuracy was lower when only CBI‐R subsection scores were used (mean AUC 0.73, Supplementary MaterialsS3 in supporting information).

Sequential feature elimination determined that all questions were required for at least one of the pairwise SVMs. SvPPA required the fewest questions to accurately differentiate it from all other groups, but still required 36 out of a total of 45 questions. A list of questions required to determine each diagnosis is in Supplementary Materials S4 in supporting information.

We then looked at the subset of participants with longitudinal data (total *n* = 1037, AD = 173, lv/other PPA = 24, PCA = 25, DLB = 56, PD = 150, bvFTD = 133, PSP = 199, CBS = 95, MSA = 24, nfvPPA = 61, svPPA = 95, MND = 2) over a mean follow‐up time of 2.7 years (SD = 2.6) and mean 2.7 visits (SD = 2.2). Over all groups, the total CBI‐R score had an annualized rate of increase of 4.9 (95% confidence interval [CI]: 4.5–5.3) with the greatest subscore increase being in everyday skills (1.9, 95% CI: 1.8–2.1; Table 2). CBI‐R progression varied widely between participants. The total CBI‐R score *improved* at the second clinic visit in 30% of participants across all groups. Plots of individual participant trajectories are in Supplementary Materials S5 in supporting information.

**TABLE 2:** Longitudinal change in CBI‐R (total and subscores as derived from principal components in previous analysis).

CBI subscore	All groups	AD	PCA	DLB	PD	MSA	bvFTD	PSP	CBS	nfvPPA	svPPA
Behavior	0.88 (0.75–1.01)	1.14 (0.90–1.38)	1.43 (0.67–2.19)	1.04 (0.43–1.66)	0.21 (0.01–0.42)	0.18 (‐0.13–0.50)	0.31 (‐0.15–0.76)	0.67 (0.21–1.13)	0.7 (0.03–1.37)	2.24 (1.71–2.76)	1.87 (1.49–2.25)
Everyday skills	1.94 (1.80–2.08)	2.38 (2.09–2.67)	3.5 (2.94–4.07)	2.51 (1.83–3.19)	0.55 (0.33–0.78)	1.78 (1.27–2.28)	2.28 (1.85–2.72)	3 (2.47–3.54)	3.16 (2.34–3.97)	3.21 (2.64–3.79)	2.33 (2.01–2.66)
Memory and orientation	0.93 (0.83–1.03)	1.42 (1.20–1.64)	2.47 (1.98–2.95)	0.88 (0.38–1.37)	0.39 (0.22–0.55)	0.07 (‐0.23–0.36)	0.87 (0.58–1.15)	0.34 (0.02–0.66)	1.28 (0.71–1.85)	1.74 (1.23–2.24)	1.28 (1.04–1.52)
Mood	0.25 (0.21–0.29)	0.42 (0.33–0.51)	0.84 (0.65–1.02)	0.34 (0.13–0.54)	0.09 (0.02–0.16)	0.08 (‐0.06–0.22)	0.21 (0.07–0.34)	0.07 (‐0.07–0.21)	0.34 (0.11–0.57)	0.61 (0.42–0.80)	0.33 (0.23–0.42)
Psychosis	0.05 (0.02–0.08)	0.15 (0.09–0.21)	0.35 (0.18–0.51)	0.03 (‐0.15–0.21)	0.08 (0.03–0.13)	−0.03 (‐0.07–0.01)	−0.21 (‐0.33—0.08)	0.06 (‐0.03–0.15)	0.19 (0.06–0.32)	0.12 (0.02–0.23)	0.04 (‐0.06–0.14)
CBI‐R total	4.93 (4.54–5.32)	6.55 (5.73–7.38)	10.17 (8.49–11.86)	5.88 (3.85–7.91)	1.63 (0.95–2.31)	2.28 (1.33–3.23)	4.23 (3.08–5.38)	5.15 (3.92–6.38)	6.46 (4.24–8.68)	9.65 (7.91–11.38)	7.14 (6.19–8.09)

*Note*: Estimates (and 95% confidence intervals) for the change in score per year of follow up. lv/mixed PPA and MND groups had insufficient participants with follow up data and are not included.

Abbreviations: AD, Alzheimer's disease; bvFTD, behavioral variant frontotemporal dementia; CBI‐R, Cambridge Behavioural Inventory short version; CBS, corticobasal syndrome; DLB, dementia with Lewy bodies; lvAD, language variants associated with Alzheimer's disease include logopenic and mixed variants of primary progressive aphasia; MND, motor neuron disease; MSA, multiple system atrophy; nfvPPA, non‐fluent variant primary progressive aphasia; PCA, posterior cortical atrophy; PD, Parkinson's disease; PSP, progressive supranuclear palsy; SD, standard deviation; svPPA, semantic variant primary progressive atrophy.

Finally, we estimated the sample size required for clinical trials for behavioral impairment in dementia, targeting a 30% reduction in symptoms as measured by the CBI‐Behavior subscore with power of 95% (Table 3). We estimated that a trial recruiting irrespective of symptom severity would require between 247 and 420 participants per arm, depending on diagnosis. Restricting recruitment to severe behavioral impairment (> 50% of symptoms present at least weekly) reduced the estimated sample size to between 33 and 40 participants per trial arm.

**TABLE 3:** Power calculations for CBI‐Behavior reduction of 30% at 6 months.

Diagnosis	Baseline mean (SD)	6 months mean (SD)	Targeted effect size (CBI‐Behavior)	Sample size (per arm)
All diagnoses	9.5 (10.5)	9.9	2.8	355
(Behavioral impairment)	24.8 (9)	24.9	7.5	40
AD syndromes (all)	8.9 (9.6)	9.6	2.7	336
(Behavioral impairment)	23.8 (8.6)	24.3	7.1	39
FTLD syndromes (all)	13.3 (12.2)	13.9	4.0	247
(Behavioral impairment)	26 (9.3)	26.0	7.8	39
αSyn syndromes (all)	5.4 (6.5)	5.6	1.6	420
(Behavioral impairment)	21.1 (6.9)	20.8	6.3	33

*Note*: CBI‐Behavior includes all CBI questions contributing to behavioral components from principal component analysis. The “behavioral impairment” subset of each group include all participants with a baseline CBI‐Behavior score > 14. Sample size calculations are calculated for two‐sample, two‐sided, *t* test with alpha 0.05 and power 95%. AD syndromes: amnestic/typical AD, logopenic/mixed primary progressive aphasia and posterior cortical atrophy. FTLD syndromes: behavioral variant frontotemporal dementia, semantic, and non‐fluent variant primary progressive aphasia, progressive supranuclear palsy, and corticobasal syndrome. αSyn syndromes: dementia with Lewy bodies, Parkinson's disease, multiple system atrophy.

Abbreviations: AD, Alzheimer's disease; CBI‐R, Cambridge Behavioural Inventory short version; FTLD, frontotemporal lobar degeneration; SD, standard deviation.

## 4 DISCUSSION

We show that a simple informant questionnaire contains sufficient information to support accurate diagnostic differentiation in six out of seven people (AUC mean 0.83), across multiple causes of dementia and movement disorder. Many behavioral manifestations occurred in multiple neurodegenerative diseases but with differential weighting on memory, everyday skills, inhibition, motivation, mood, and sleep.

We do not recommend replacing clinical assessment, examination, and investigation by a simple informant questionnaire. Instead, we emphasize the importance and relevance of information available from a collateral history, and the capacity for a simple, safe, cheap tool like the CBI‐R to aid diagnosis. We do not advocate clinical use of the machine learning model trained on the CBI‐R. Rather, we highlight that the performance of a short informant questionnaire is similar to the diagnostic accuracy of advanced cognitive tests^49^, ^50^ and neuroimaging methods.^51^, ^52^, ^53^, ^54^, ^55^, ^56^, ^57^, ^58^, ^59^ This high accuracy from a simple tool provides a high benchmark against which to evaluate more complex, invasive, or expensive tools.

Other caregiver questionnaires or caregiver interview–based outcomes are used in clinical and trials assessments. The NPI^16^ is widely used and can help discriminate between participants with dementia and normal cognition (AUC 0.76)^60^ and between some diseases (e.g., between AD and FTD with the FTD module).^60^ The Frontal Behavioral Inventory (FBI)^61^ can distinguish participants with bvFTD from those with other dementia diagnoses.^62^, ^63^ The Informant Questionnaire on Cognitive Decline in the Elderly can differentiate participants with and without dementia,^64^ but is less accurate between dementia diagnoses.^65^ Item‐level rather than summary scores of these other tests might also improve diagnostic accuracy.

The pattern of responses to the 45 individual questions varied—and covaried—within questionnaire subdomains and diagnoses. For example, episodic memory loss was reported in the classical, amnestic presentation of AD but also other AD subtypes, DLB, and even some people with bvFTD. Questions that reflect overall function (“everyday skills”) underpinned a distinct component, suggesting functional impairments do not reflect a single cognitive or motor domain. Diseases that present with both cognitive impairment and a movement disorder (e.g., PSP, CBS, and DLB) were associated with marked difficulty with everyday skills. Behavioral disinhibition (inappropriate humor, anger outbursts, embarrassing behavior) were manifest to a degree across most diseases, including AD^13^, and not only bvFTD, which includes such behaviors as diagnostic criteria. In contrast, hyperorality and repetitive behavior was more restricted to FTLD‐associated syndromes (bvFTD, svPPA, and PSP^42^). Apathy was distinguished from anhedonia and depression (motivation and mood‐related questions underpinned different components)^66^, ^67^ and was highly prevalent in all neurodegenerative diseases as reported previously.^66^, ^68^, ^69^, ^70^, ^71^

There are limits to the diagnostic utility of the CBI‐R. Relevant physical signs, such as the gaze palsy of PSP, may not be noticed by patients and caregivers and require neurological examination. Complex psychiatric phenotypes such as hallucinations or delusional beliefs may be mischaracterized or misinterpreted by untrained observers. Nonetheless, informant observations capture other manifestations that correlate highly with a diagnosis based on a full examination, history, and investigation. Informant questionnaires may add value to the diagnosis, or facilitate the clinician in eliciting a full and relevant history. For example, PD and PSP can be difficult to distinguish clinically,^72^ but the CBI‐R revealed behavioral impairments which distinguish them.^73^ Another limitation is that some cognitive impairments may be relatively specific to a diagnostic group (for example severe visuospatial impairments in PCA) but only manifest in non‐specific endorsements on the CBI‐R. A clinical assessment allows for additional questions to improve accuracy.

Our data highlight the distinction between diagnostic and monitoring tools, which also applies to imaging, fluidic, and cognitive biomarkers. Although the CBI‐R total and subscores worsened over time, there was wide variability within all diagnostic groups^15^.The CBI‐R total score improved between the first and second clinic visit in 29.9% of participants (before typically worsening again in those who attended a third visit). There are several interpretations of this result. It is possible that after the first visit, there was therapeutic benefit from both pharmacological^74^, ^75^ and non‐pharmacological^76^ treatment even in the absence of disease‐modifying treatments. Alternatively, caregivers may interpret the questionnaires differently after a diagnosis. Finally, disease progression may mitigate some behaviors, only later to be replaced by others. For example, in bvFTD, the degree of disruption from challenging behaviors may temporarily improve with the emergence of akinesia or aphasia.

The use of informant data complements patient‐reported outcomes as trial endpoints.^77^ Patient ‐reports can be difficult to interpret in participants with cognitive impairment, aphasia, or anosognosia.^78^ This does not mean that behavioral impairments are “asymptomatic” and unrelated to quality of life. Memory loss or severe behavioral disinhibition may lead to reduced independence, social withdrawal, and eventually institutionalisation.^79^ Moreover, self‐report questionnaires assume the meaningfulness of responses, which is undermined by FTD.^78^ Proxy reporting from caregiver reports such as in the CBI‐R can measure these relevant and burdensome clinical features and support trial outcomes even if not patient reported directly. It is important that the same individual completes the CBI‐R on longitudinal studies, as people with different roles (spouse, child) seem to differ in the severity of endorsements. Further research on the factors that affect informant perspectives of neurodegenerative disease is needed, including socioeconomic circumstances, ethnicity, cohabitation, and co‐morbidity.

This transdiagnostic approach could facilitate the development of treatments and clinical trial design. For example, a basket trial^80^ targeting behavioral changes could recruit patients with high endorsements on CBI‐R questions reflected by the behavioral impairment components, irrespective of their underlying diagnosis. Of course, fewer patients would be eligible so the benefit of such trials will depend on the prevalence of their severe behavioral impairments. Transdiagnostic clinical features, or symptom profiles, may reflect a common underlying mechanism.^81^, ^82^ However, our data in themselves do not prove such mechanistic commonality, in functional anatomy, pathophysiology, or molecular pathology. For example, psychosis may have distinct mechanisms in AD, DLB, and FTD. Further research would be needed to confirm whether transdiagnostic symptom profiles share common mechanisms, as the basis for common treatment targets.^81^, ^82^ An additional consideration is that minimal clinically important differences are not widely established for caregiver‐rated endpoints so our power calculations were based on a 30% reduction in symptoms, similar to the efficacy of levodopa and dopamine agonist therapy in PD and serotonin reuptake inhibitor therapy in depression.^83^, ^84^, ^85^, ^86^, ^87^, ^88^

Our study has other limitations. First, we calibrate the CBI performance against a clinical diagnosis. This is informed by relative and caregiver collateral history, which is likely to have contained information that was reflected in their completion of the CBI‐R form. Only a subset had neuropathological confirmation, with clinico‐pathological correlations in line with published reports.^40^, ^45^, ^89^, ^90^, ^91^, ^92^ We are not comparing the CBI‐R response pattern to the molecular pathology of each patient. The relative contribution of each question to the differential diagnosis in the SVM is difficult to illustrate. However, the principal components shown in Figure 1 indicate the degree to which endorsements are differentially endorsed, by diagnosis. We did not test the ability of the CBI‐R to distinguish dementia “mimics” such as psychiatric disorders or attentional memory problems, in part because of a lack of operational definitions.^40^, ^45^, ^89^, ^90^, ^91^, ^92^, ^93^ Second, our data are derived from a single site, albeit with a large catchment area across the East of England. This catchment area is diverse in socioeconomic status but not ethnicity. Language and ethnicity variation in informant reports requires further examination and is likely to require more than simple translation of the questions.^94^ Our focus on CBI‐R score at diagnosis also meant that patients who develop dementia later in their illness (e.g., PD followed by dementia) may be underrepresented. Third, SVM learning is a relatively simple classification technique and more advanced machine learning techniques may better model our data.^51^ We did not use a separate dataset for testing, limiting the external validity of our results, although we mitigate this by the large size of the cohort. Fourth, CBI‐R performance was not compared to other caregiver questionnaires, such as the NPI or FBI, or to measures of patient quality of life or caregiver burden. This comparative performance has been reported elsewhere,^20^, ^95^, ^96^ and was not required to address our hypotheses.^81^, ^82^ Finally, our results are not corrected for disease stage or severity. In most patients we used the caregiver questionnaire at the diagnostic visit. However, patients may face long diagnostic delays^97^, ^98^ and our results could be confounded by group‐wise variation in disease stage. Patients may have been at different stages at diagnosis although they are in the similar health‐care context of their initial secondary care assessment.

In summary, we have shown that informant data, giving relative or caregiver perspectives on the behavior of people with dementia and movements disorders, contains sufficient information for differential diagnosis in six out of seven cases. Symptom profiles are not unique to each disease, but rather they are expressed to varying degrees across multiple neurodegenerative diseases. William Osler reportedly said: “Listen to your patient, he is telling you the diagnosis.” Our results suggest a corollary, that as clinicians we should also “Listen to your patient's relative, they are also telling you the diagnosis.” Such caregiver‐based information can also be elicited from a simple structured report format like the CBI‐R, with remarkable accuracy for differentiating dementias and movement disorders.

## CONFLICT OF INTEREST STATEMENT

The authors have no conflicts of interest to report related to this work. Unrelated to this work, AGM: none; LB: none; MC: has received grants from the Evelyn Trust; CHWG: has received grants from Cure Parkinson's, Parkinson's UK, and the Rosetrees Trust; honoraria from GSK and Elsevier; consultancy fees from Evidera, Inc.; travel stipends from the World Parkinson's Coalition, Parkinson's UK, and the European Cooperation in Science and Technology; KAT: none. TR: none; RB: has received grants from National Institute for Health and Care Research, UK, Michael J. Fox Foundation, Rosetrees Trust, European Union, Medical Research Council, UK and Wellcome Trust; honoraria from Novo Nordisk for talks, royalties from Wiley and Nature‐Springer, and is a consultant for Aspen Neuroscience, Novo Nordisk, Bluerock Therapeutics, and UCB. JOB: has received honoraria for work as DSMB chair or member for TauRx, Axon, Eisai, and Novo Nordisk, and has acted as a consultant for Biogen and Roche, and has received research support from Alliance Medical and Merck. JBR: non‐remunerated trustee of the Guarantors of Brain, Darwin College, and the PSP Association; provides consultancy to Alzheimer Research UK, Asceneuron, Astronautx, Biogen, Curasen, CumulusNeuro, UCB, SVHealth, and Wave; and has research grants from AZ‐Medimmune, Janssen, Lilly as industry partners in the Dementias Platform UK. Author disclosures are available in the supporting information.

## CONSENT STATEMENT

Participants provided written informed consent or, if they lacked capacity to consent, their next of kin was consulted as established in UK law. During this consultee process, the next of kin of a potential participant advises the researcher on what the participant's wishes and feelings on participating in the study would have been if they were able to consent for themselves. All data were fully anonymized before analysis.

## Supporting information



Supporting Information

Supporting Information
