# Supplementary material for: Caregiver perspectives enable accurate diagnosis of neurodegenerative disease
Source: Alzheimers Dement. 2024 Nov 19;21(1):e14377. doi: 10.1002/alz.14377 (PMC11772714; doi:10.1002/alz.14377)
Supplement: Supplementary file 1 — Supporting Information [file ALZ-21-e14377-s002.docx]

**Supplementary Materials 1: Clinicopathological correlation in subset of participants with neuropathology**

|  |  | **Clinical diagnosis** | | | | | | | | | | | |
| --- | --- | --- | --- | --- | --- | --- | --- | --- | --- | --- | --- | --- | --- |
| **Neuropathological diagnosis** |  | **AD** | **bvFTD** | **CBS** | **DLB** | **lv/mixedPPA** | **MND** | **MSA** | **nfvPPA** | **PD** | **PCA** | **PSP** | **svPPA or SD** |
|  | **AD** | **21** | 4 | 10 |  | 3 |  | 1 | 10 |  | **5** |  | 1 |
|  | **αSyn-LBD** |  |  | 1 | **4** |  |  |  |  | **20** |  |  |  |
|  | **αSyn-MSA** |  |  |  |  |  |  | **4** |  |  |  | 1 |  |
|  | **FTLD-Tau-Picks** |  | **8** | 1 |  |  |  |  |  |  |  |  | 2 |
|  | **FTLD-Tau-PSP** |  | **2** | 9 |  |  |  | 1 | 1 | 1 |  | **76** | 1 |
|  | **FTLD-Tau-CBD** | 1 | **3** | **21** |  | 1 |  |  | 2 |  |  |  | 1 |
|  | **FTLD-Tau-other** |  | **3** |  |  |  |  |  |  |  |  |  | 1 |
|  | **FTLD-TDP43** | 2 | **12** | 3 |  |  |  |  | 3 |  |  |  | **12** |
|  | **FTLD-U** | 1 | **14** |  |  |  | **1** |  | 2 |  |  | 1 | **7** |
|  | **FTLD-FUS** |  | **1** |  |  |  |  |  |  |  |  |  |  |
|  | **Mixed** | 3 | 4 | 7 | 2 |  |  | 1 |  |  | 1 | 2 | 1 |
|  | **DLDH** |  | 3 |  |  |  |  |  | 1 |  |  |  |  |
|  | **Other (non-neurodegenerative)** |  | 3 | 2 |  |  |  |  |  |  |  |  |  |
|  | **Total** | 28 | 57 | 54 | 6 | 4 | 1 | 7 | 19 | 21 | 6 | 80 | 26 |

Abbreviations: AD=Alzheimer’s disease, bvFTD=behavioural variant frontotemporal dementia, CBS=corticobasal syndrome, DLB=dementia with Lewy bodies, lvPPA=logopenic variant primary progressive aphasia, MND=motor neuron disease, MSA=multiple systems atrophy, nfvPPA=non-fluent variant primary progressive aphasia, PD=Parkinson’s disease, PCA=Posterior Cortical Atrophy, PSP=Progressive supranuclear palsy, svPPA=semantic variant primary progressive aphasia, SD=semantic dementia. αSyn=alpha-synucleinopathy, LBD=Lewy Body Disease, FTLD=frontotemporal lobar degeneration,FTLD--U=Frontotemporal lobar degeneration with ubiquitin-positive inclusions (all but one of these diagnoses were made before the use of TDP43 immunohistochemistry at our centre). DLDH= dementia lacking distinctive histopathologic features.

These are the contemporaneous neuropathological disease made at time of post-mortem. Historical cases have not been re-examined with use of current immunohistochemistry and neuropathological diagnostic criteria. Practice into the brain bank has also varied over the ~20 years and participants with atypical presentations (even if meeting diagnostic criteria) are more likely to have been recruited.

**Supplementary Materials 2: Itemised CBI-R responses by group**

**CBI – Memory and Orientation**

**
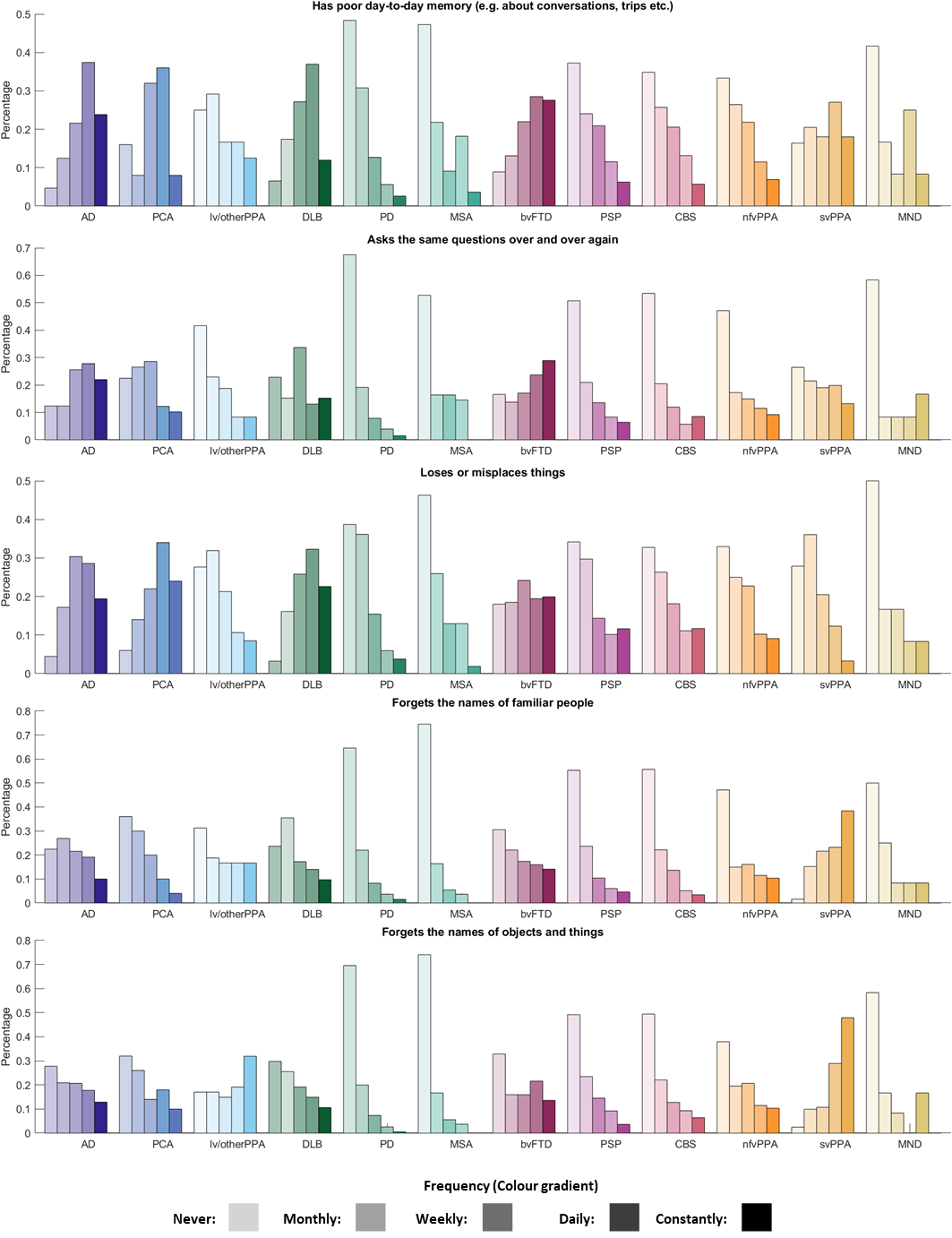
**

**CBI – Memory and Orientation Continued**

**
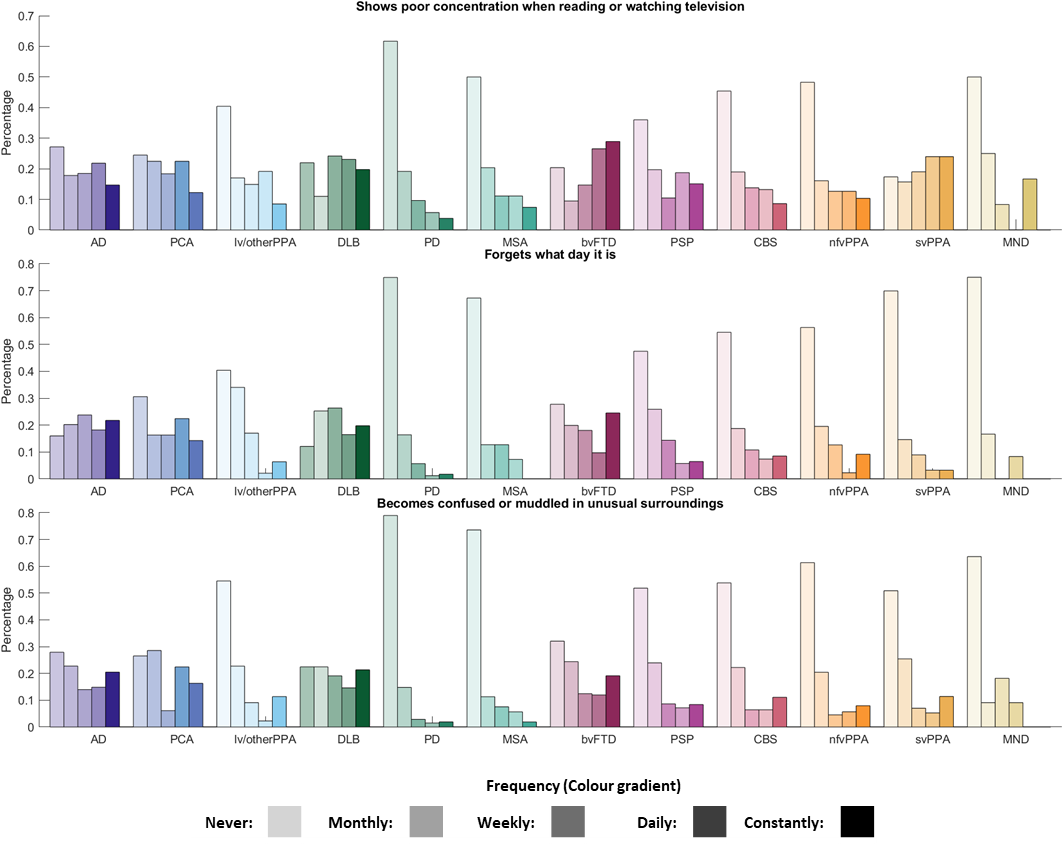
**

**CBI – Everyday Skills**

**
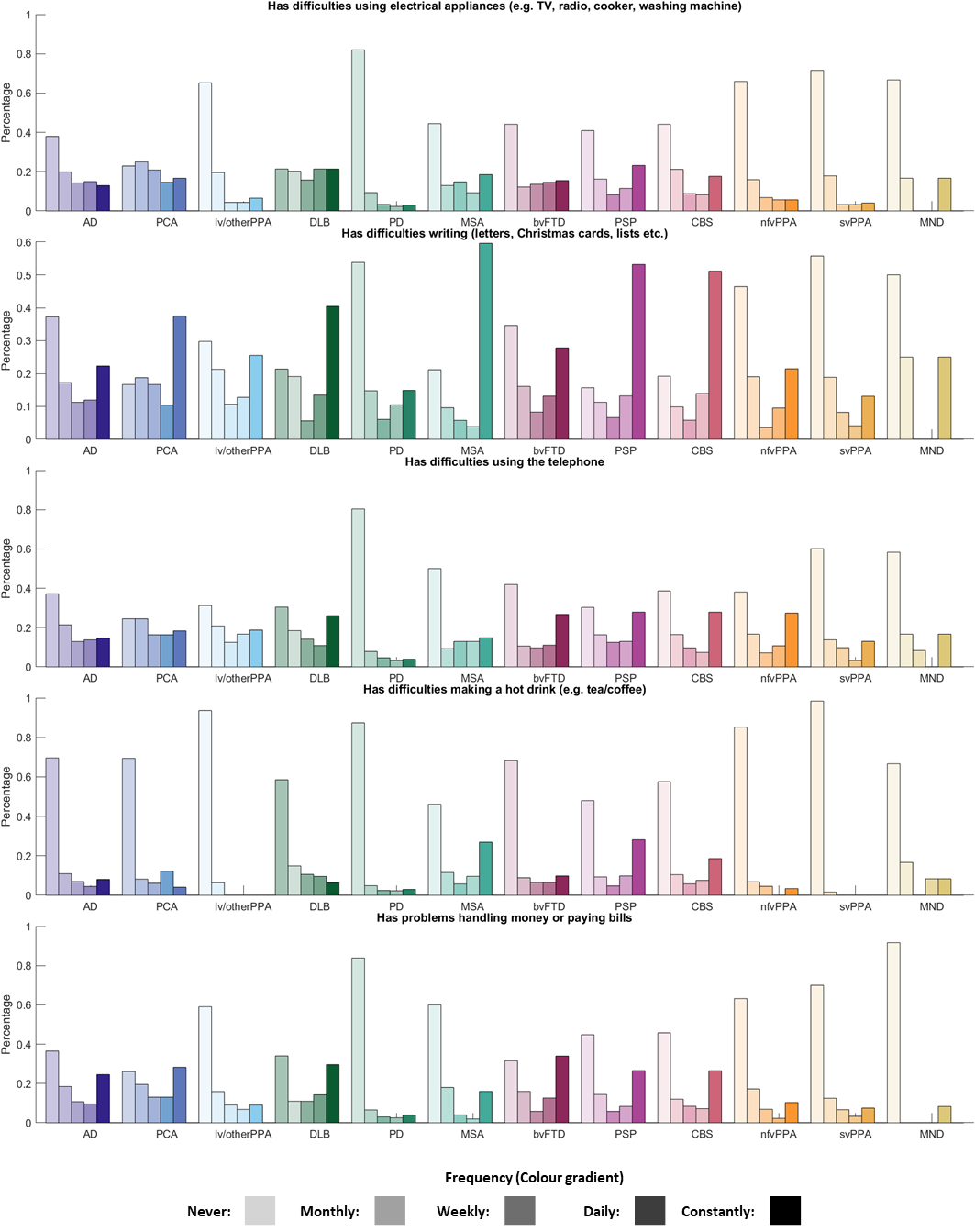
**

**CBI – Self Care**

**
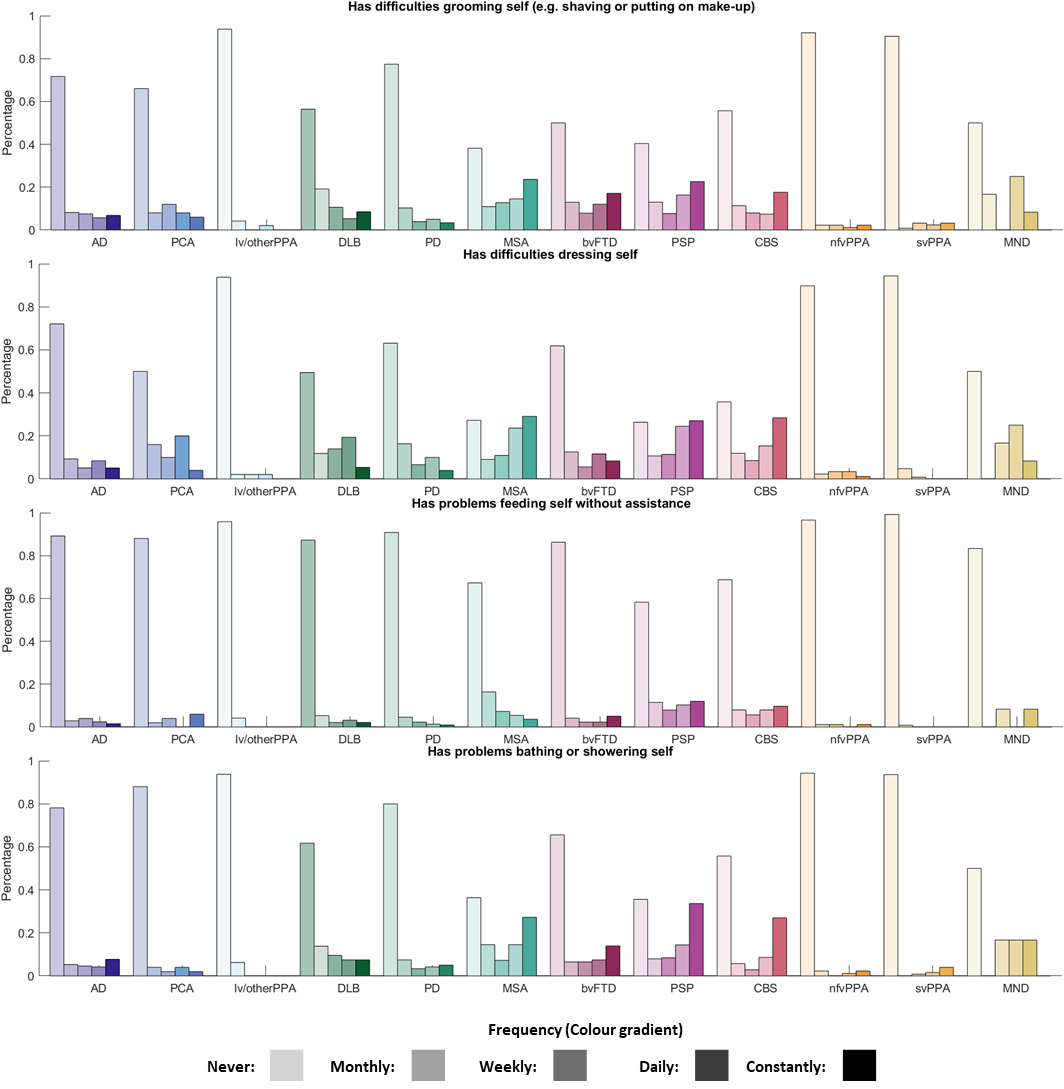
**

**CBI – Abnormal Behaviour**

**
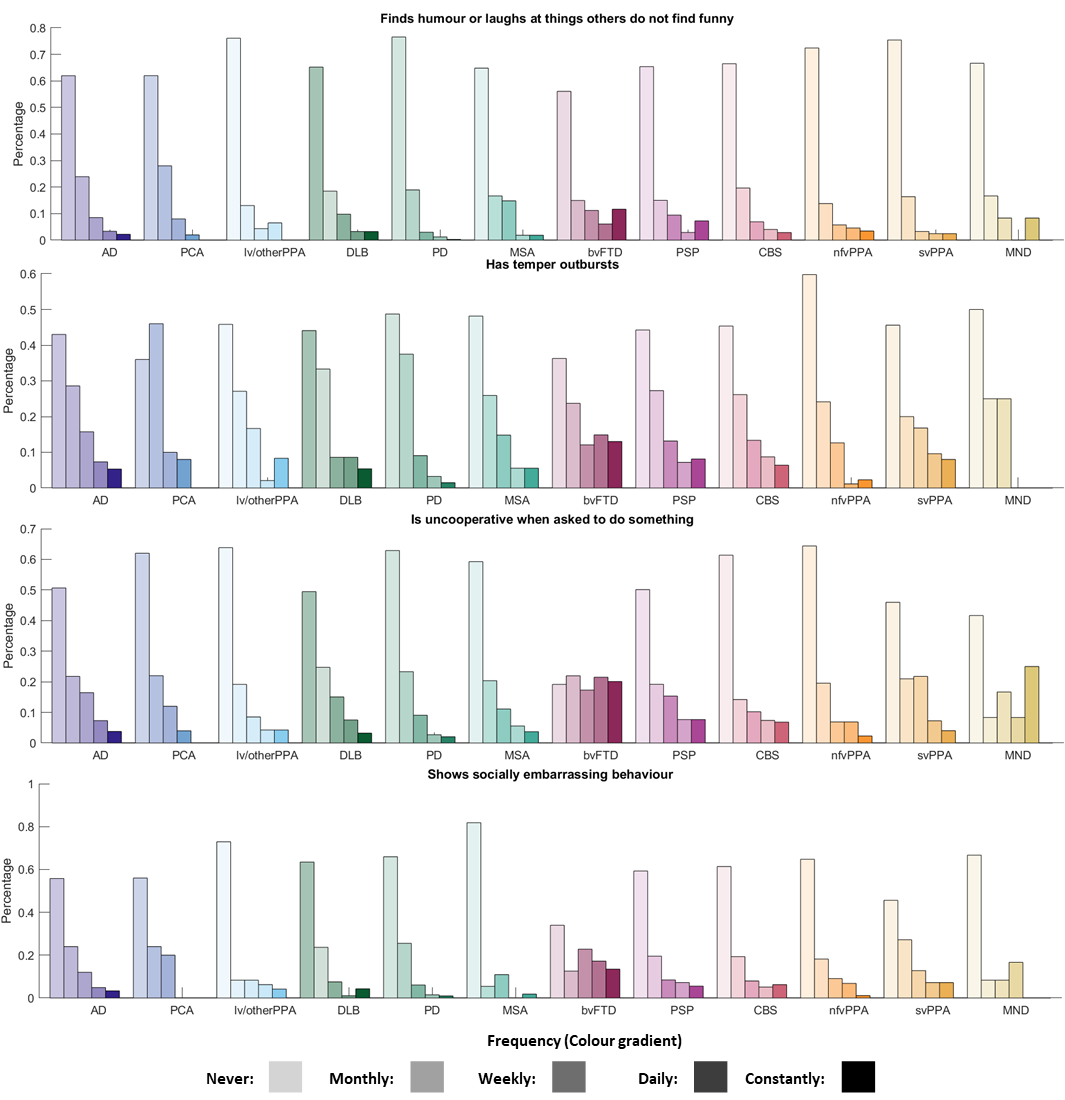
**

**CBI – Mood**

**
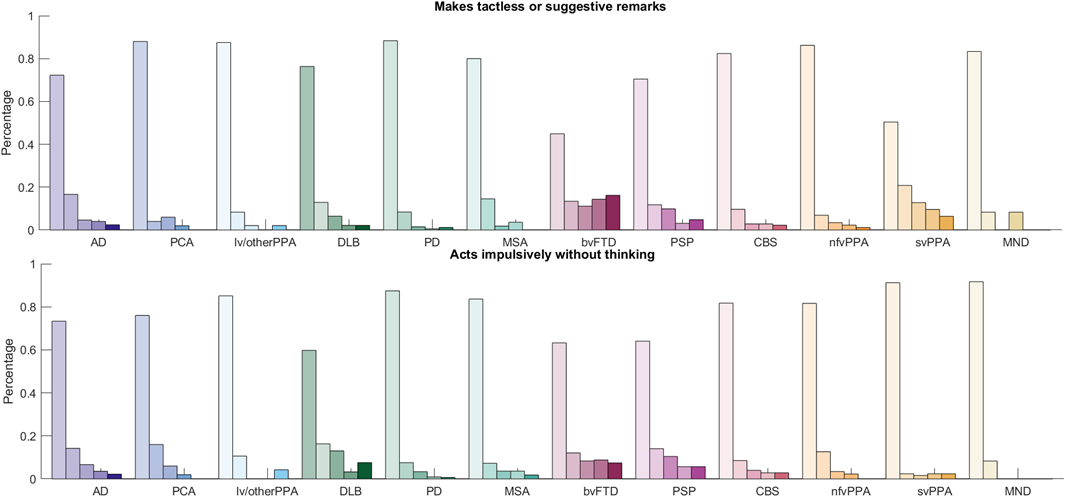
**

**CBI – Sleep**

**
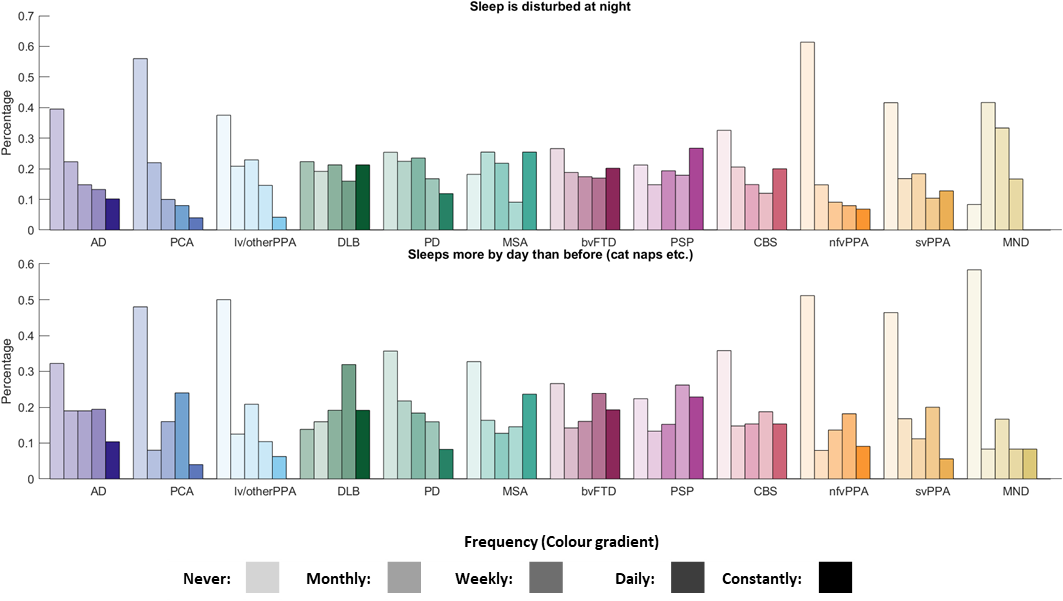
**

**CBI – Mood**

**
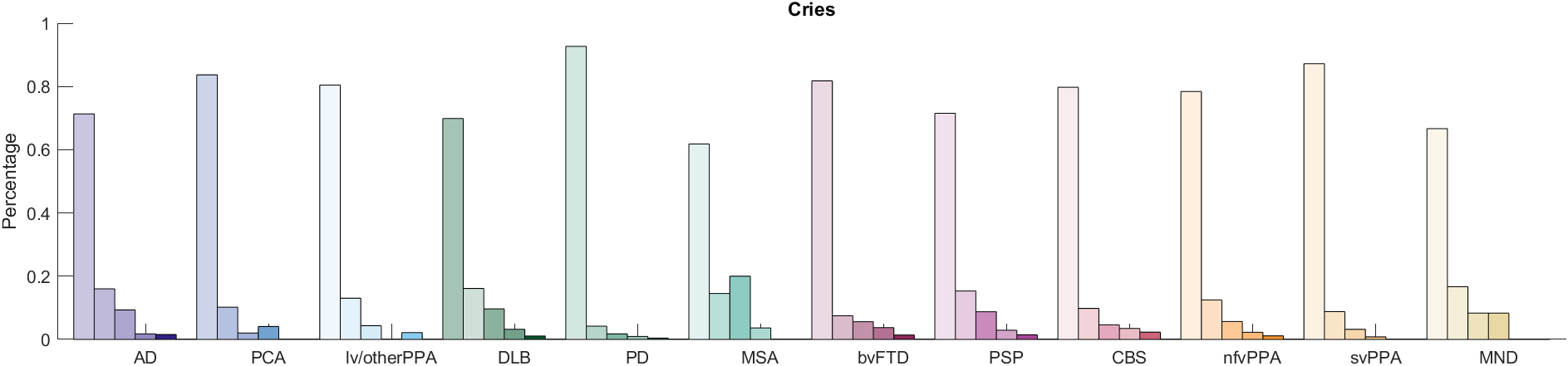

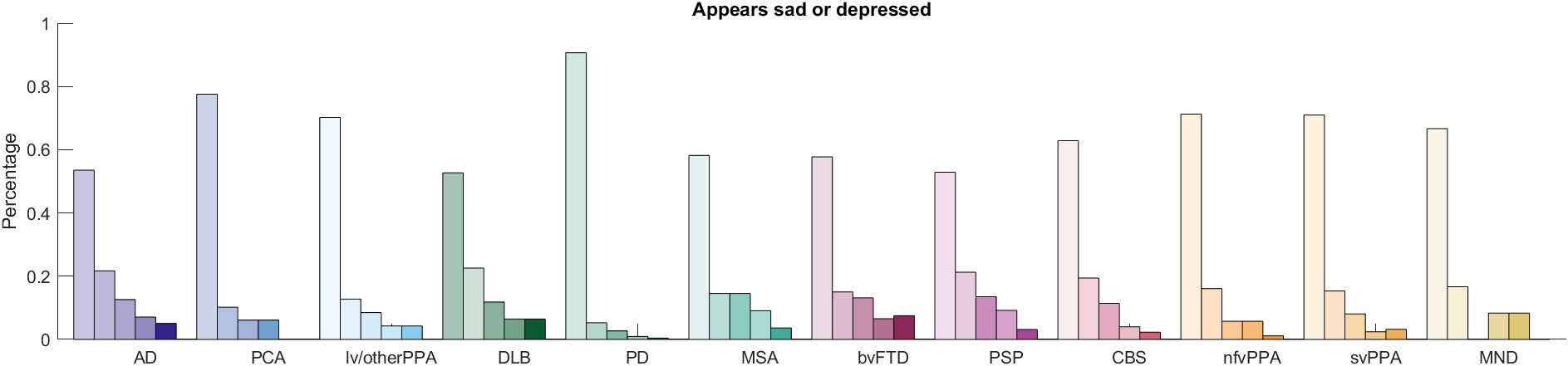

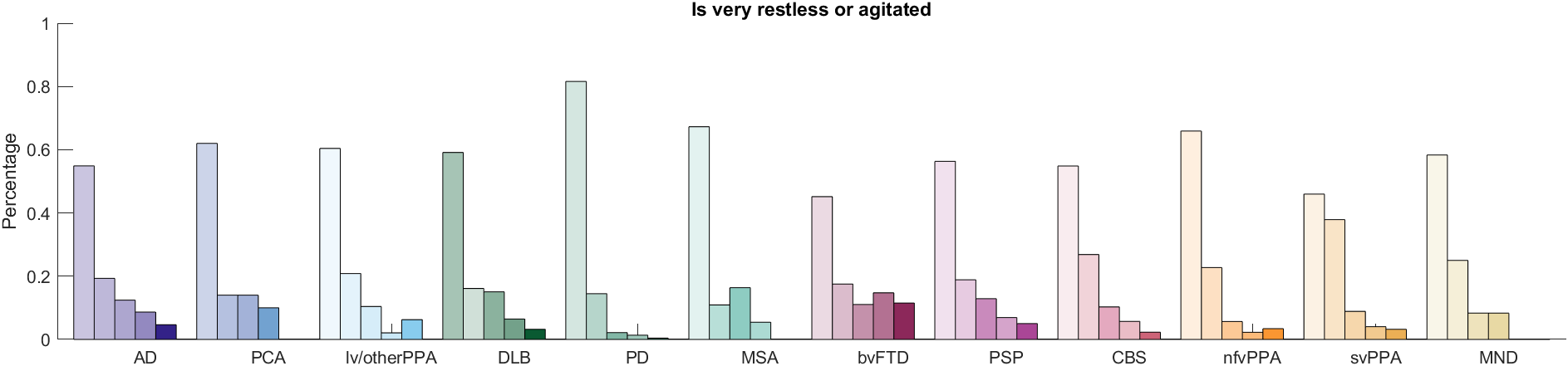

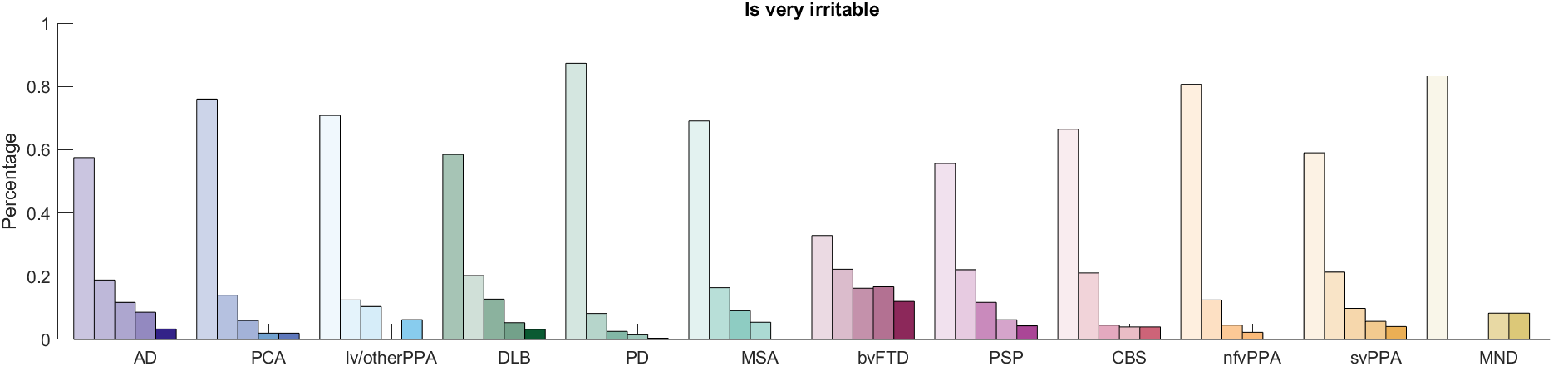

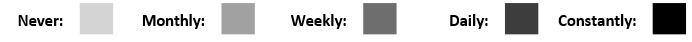
**

**Frequency (Colour gradient)**

**CBI - Beliefs**


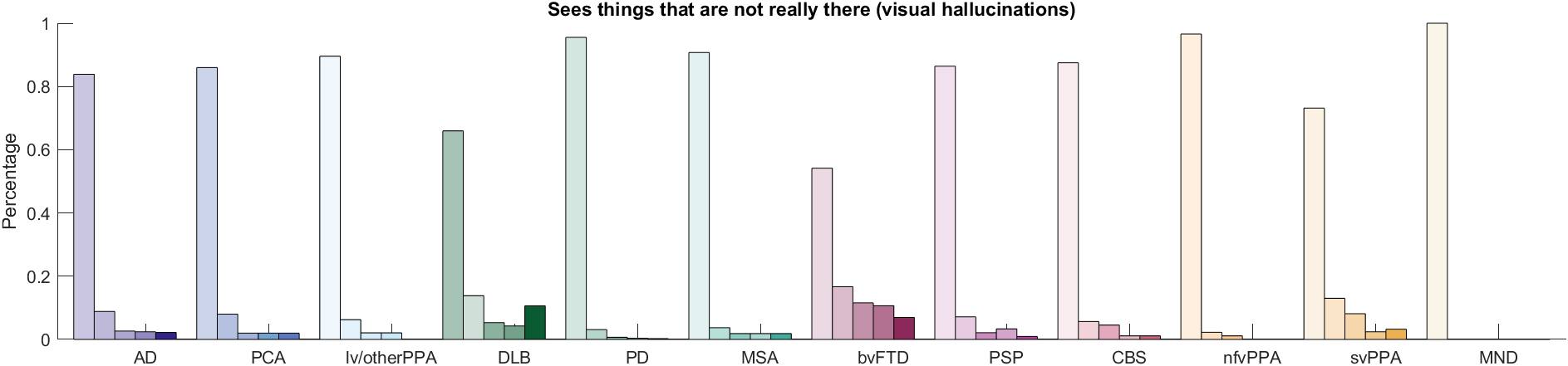

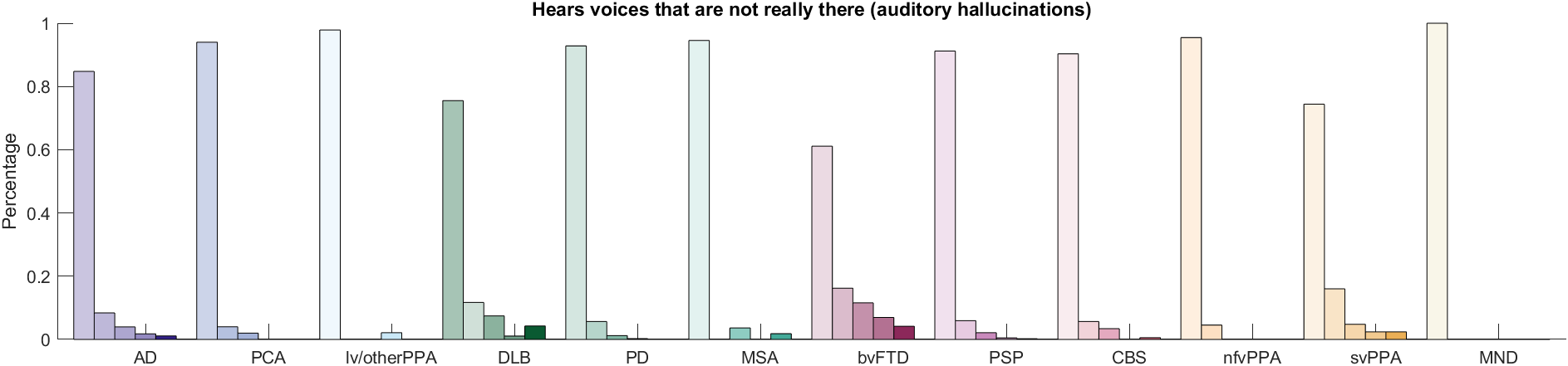

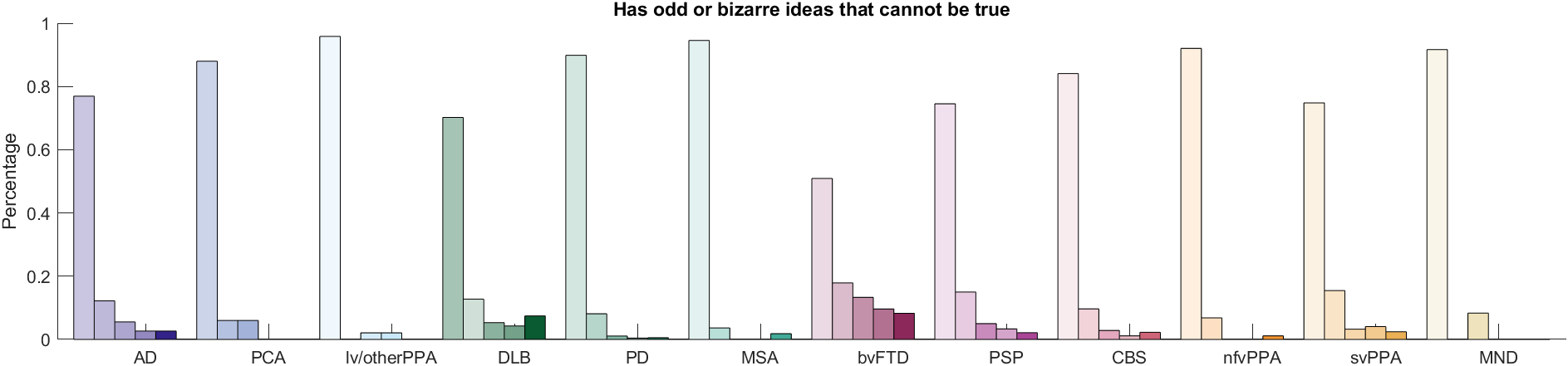

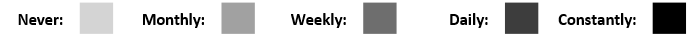


**Frequency (Colour gradient)**

**CBI – Eating Habits**

**
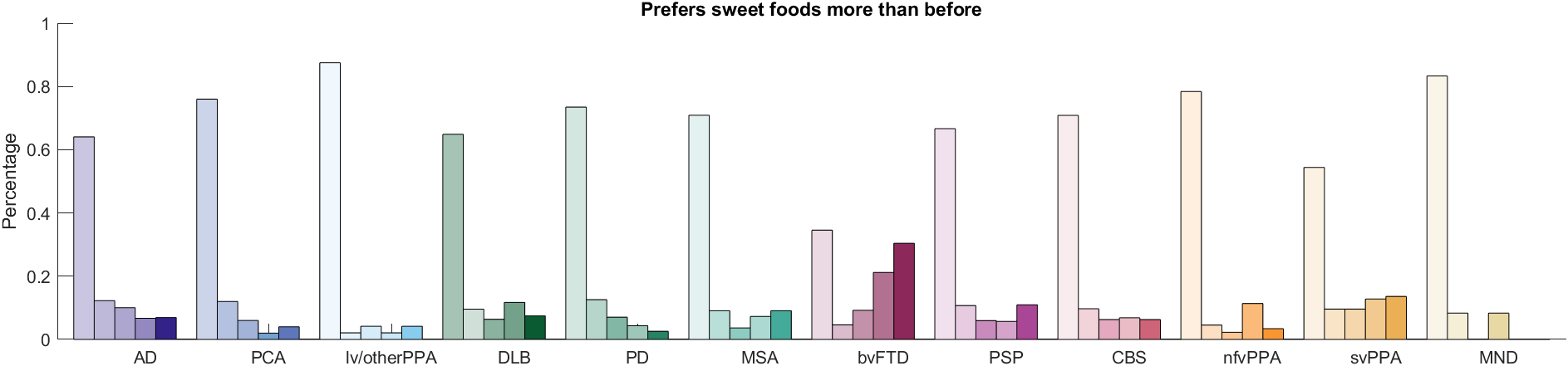

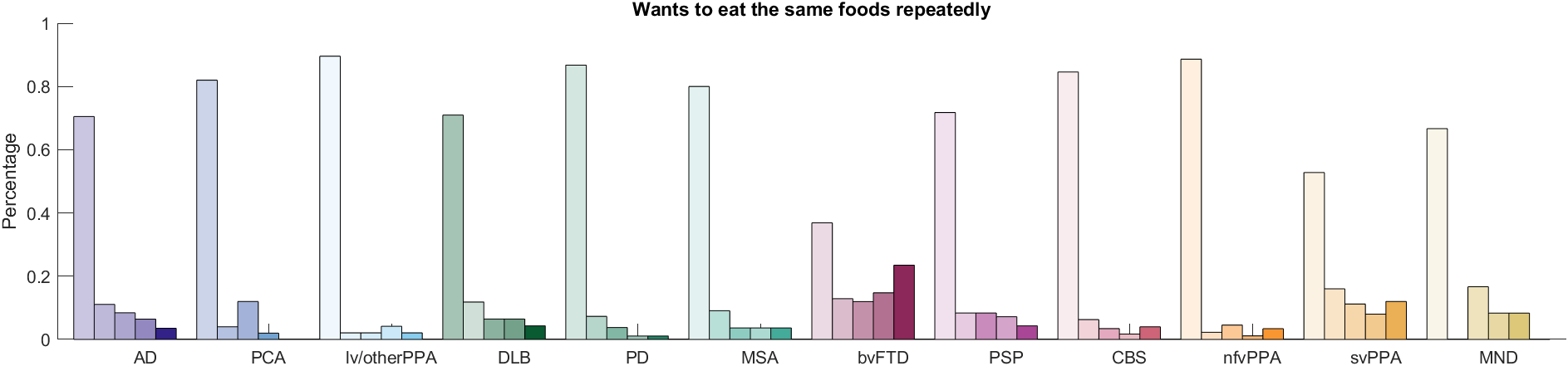

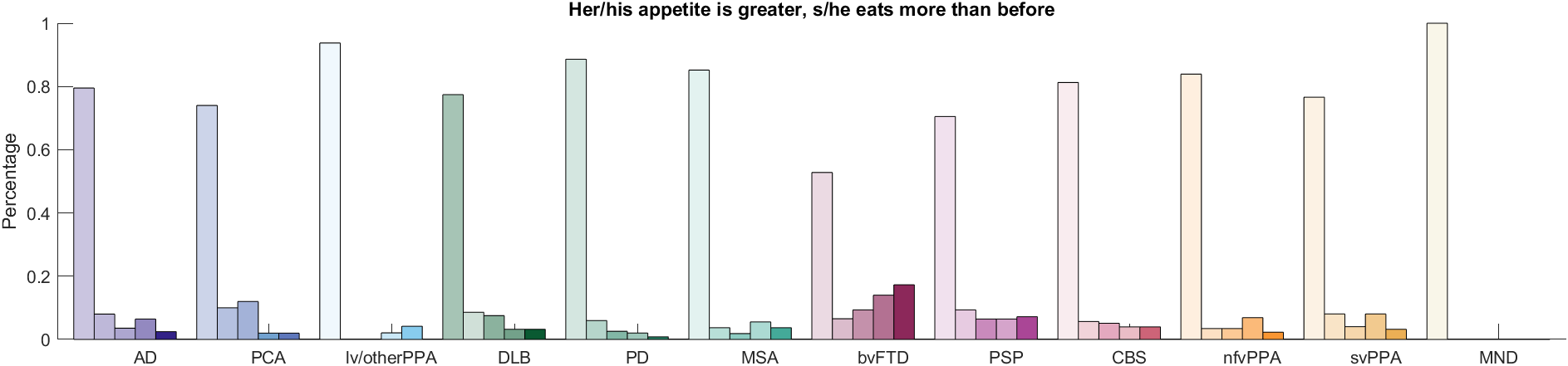

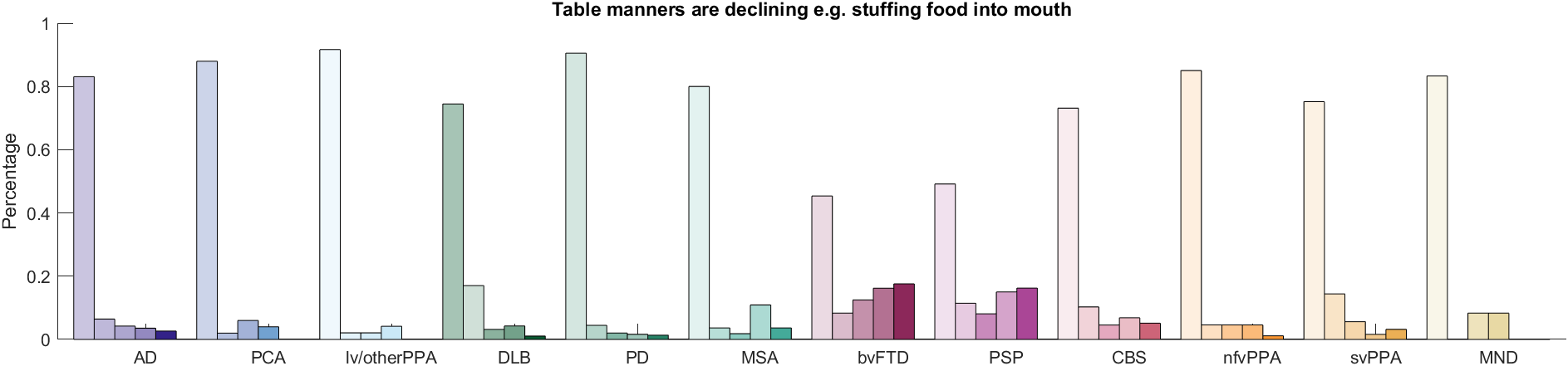

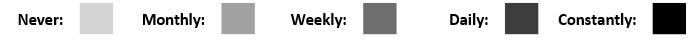
**

**Frequency (Colour gradient)**

**CBI - Stereotypic and Motor Behaviours**

**
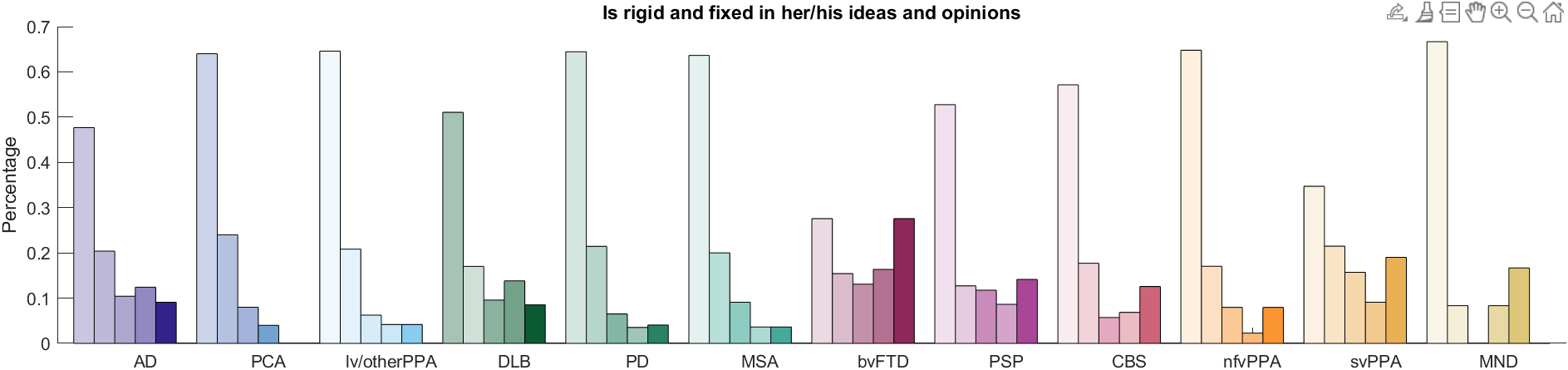

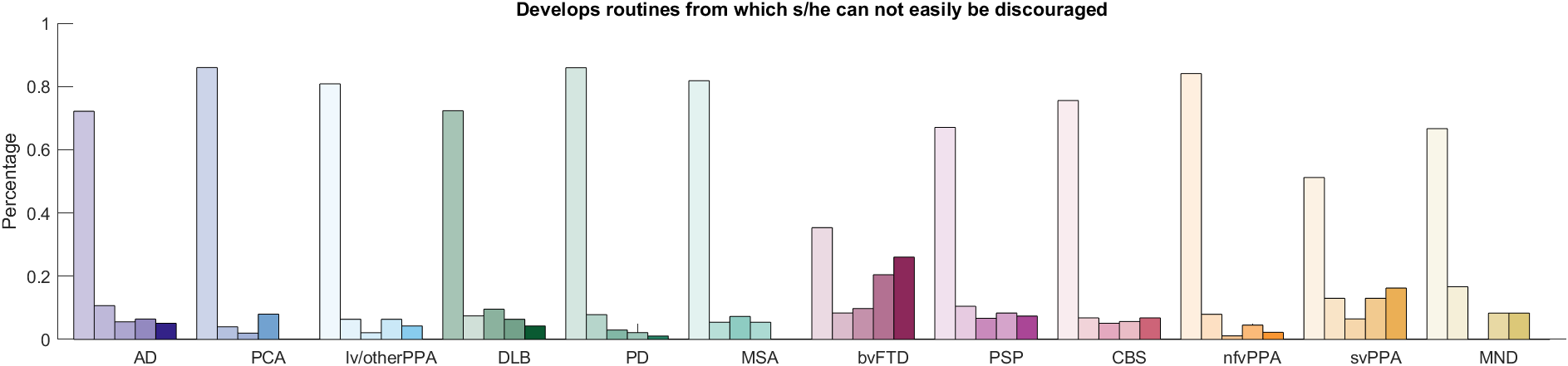

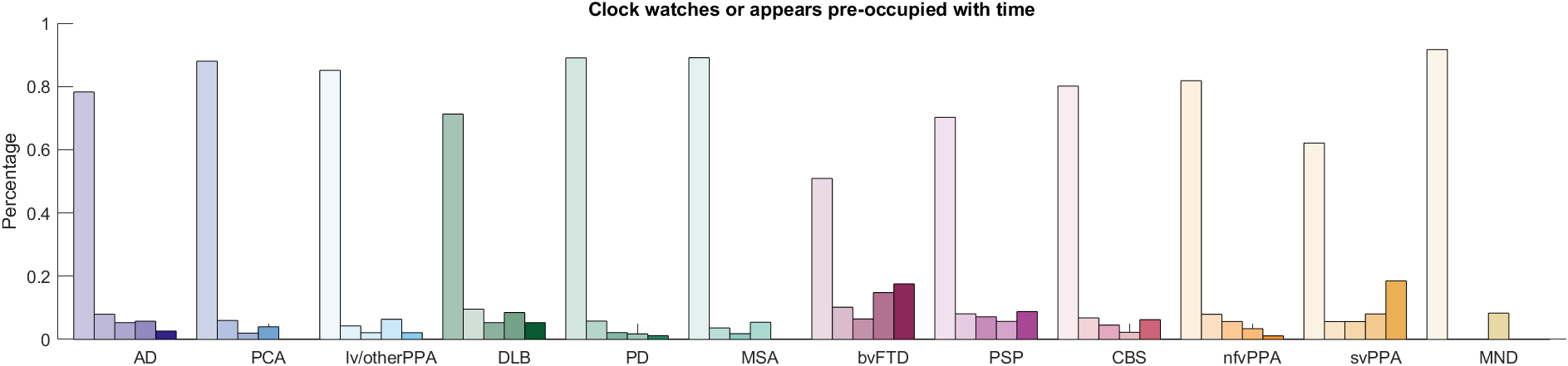

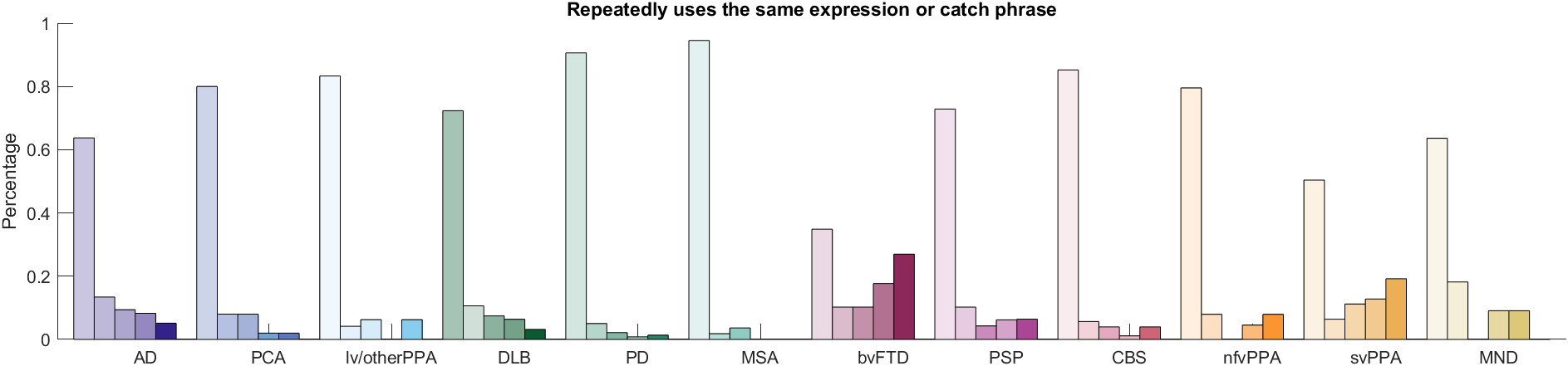

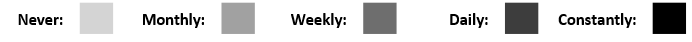
**

**Frequency (Colour gradient)**

**CBI – Motivation**

**
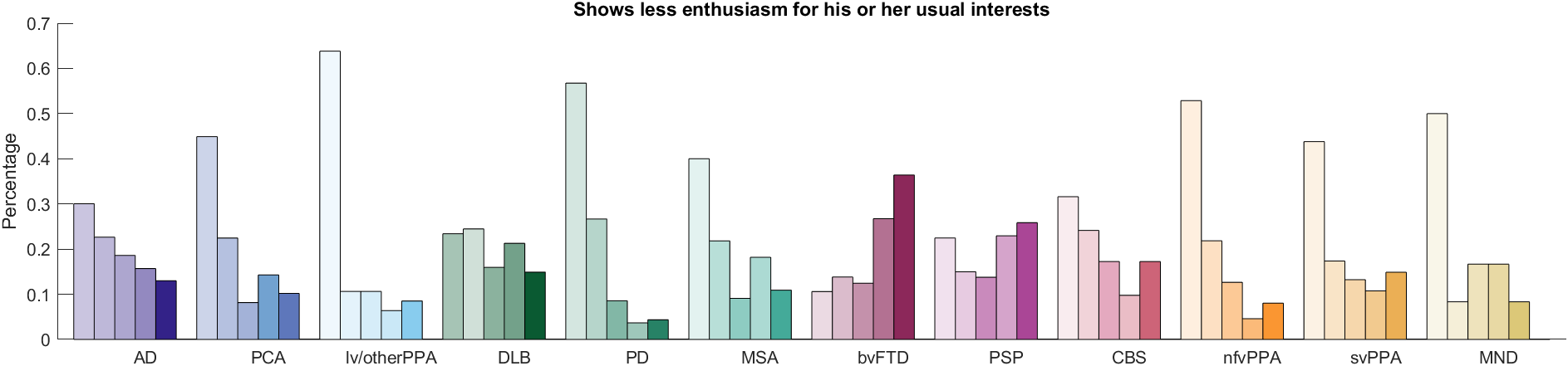

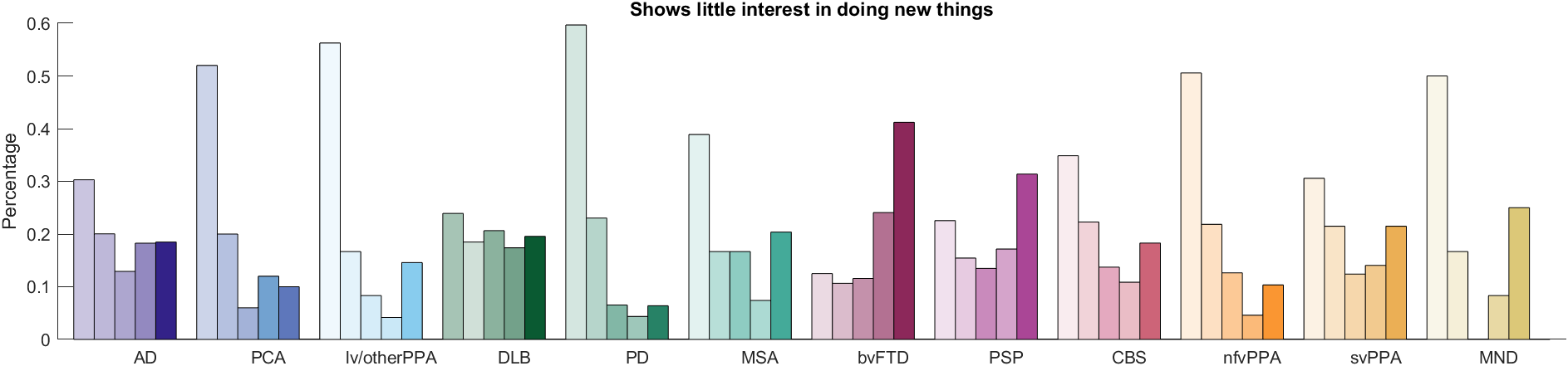

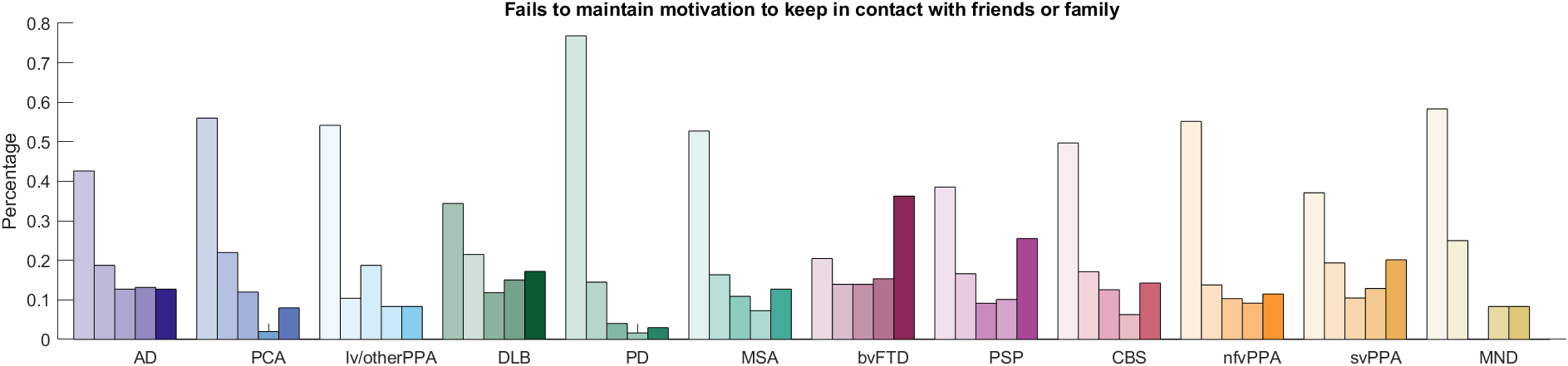

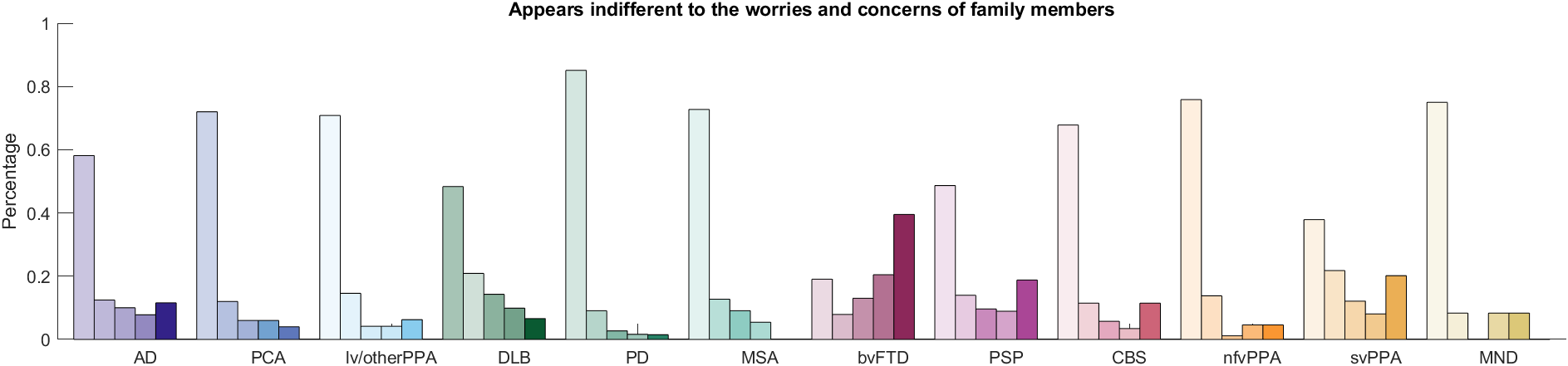

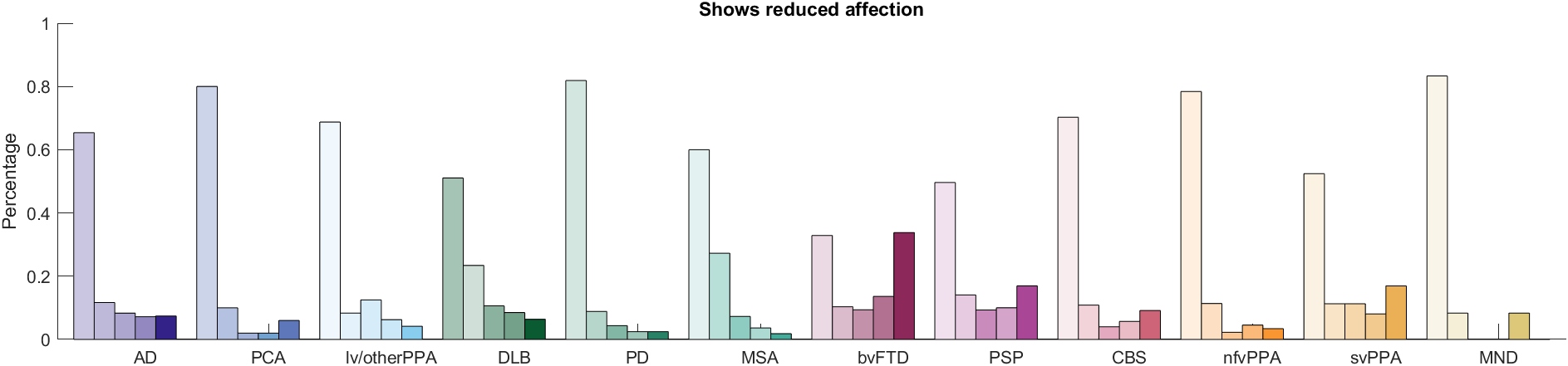

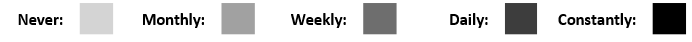
**

**Frequency (Colour gradient)**

**Supplementary Materials 3: Support Vector Machine Learning using CBI-R subsection scores**

**
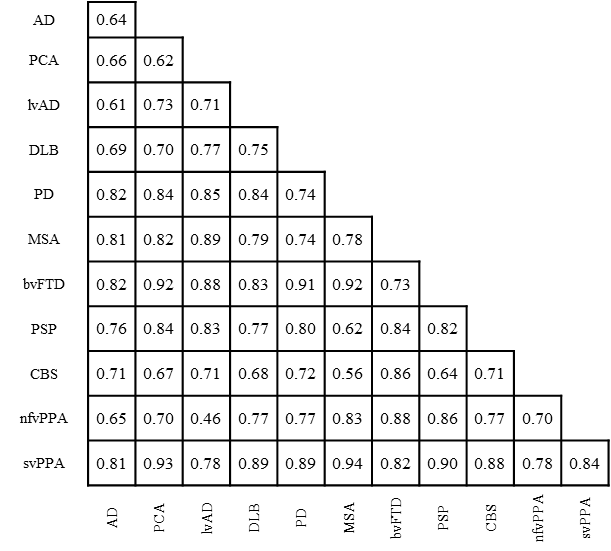
**

Area under the ROC curve (AUC) scores for all pairwise comparisons (from one vs one SVM) using subscale rather than item-level responses. The diagonal values represent the AUC scores for the one vs all SVM. The input data for these models was the CBI-R subsection totals (Everyday skills, self care, abnormal behaviour, mood, abnormal beliefs, eating habits, sleep, motor behaviour and motivation).

**Supplementary Materials 4: Support Vector Machine Sequential Feature Elimination Results**

**
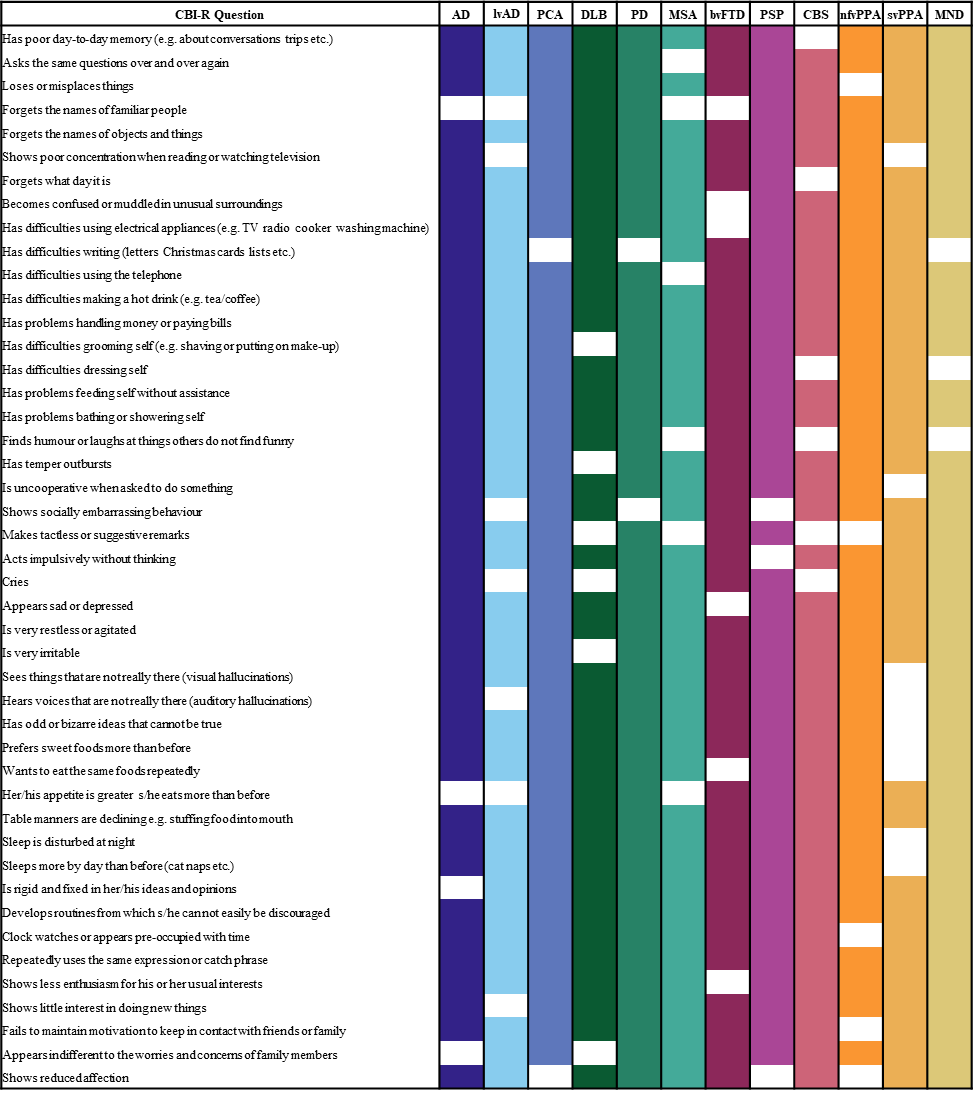
**

**Supplementary Materials 5: Individual CBI-R follow up score by diagnosis**

**
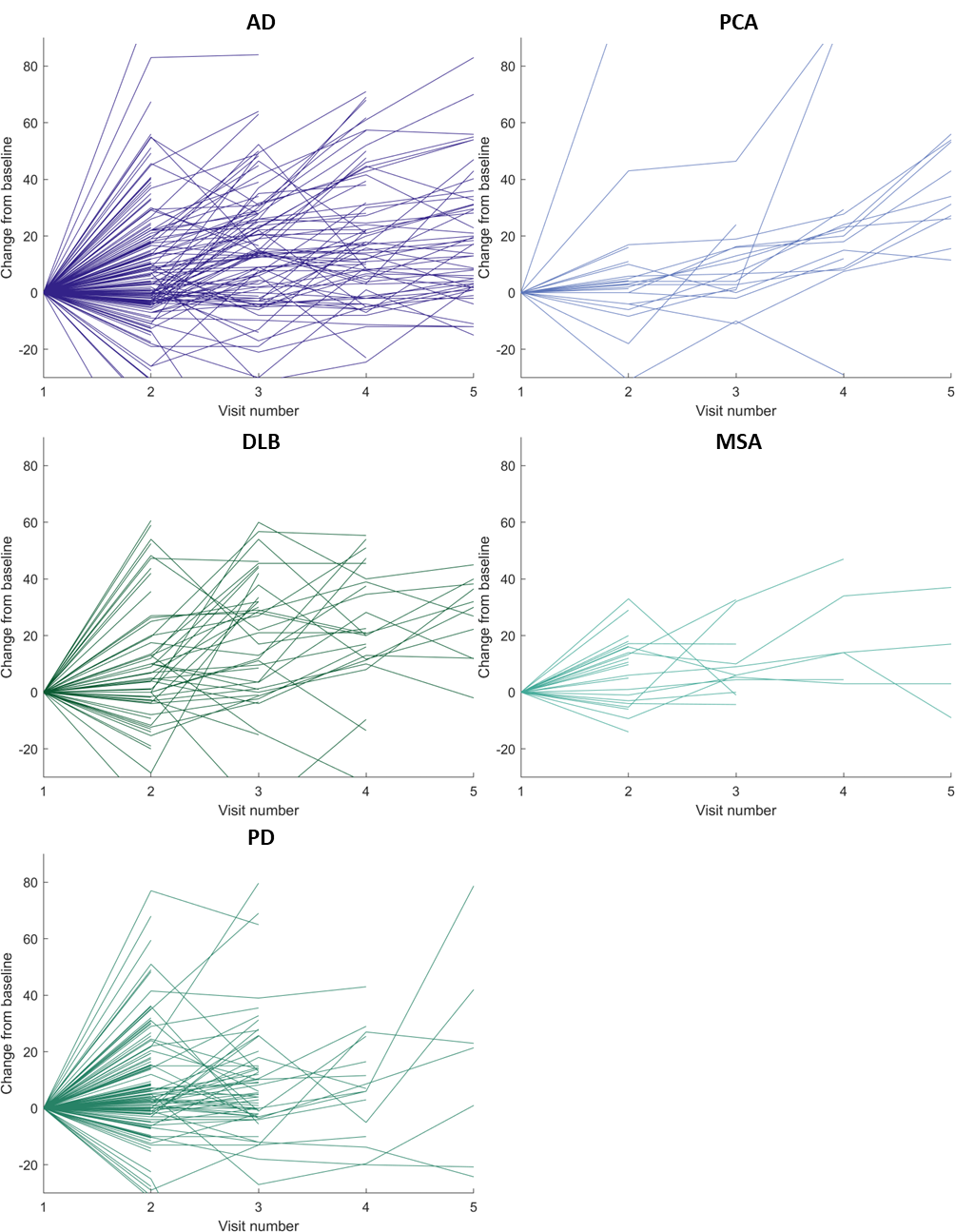
**

**
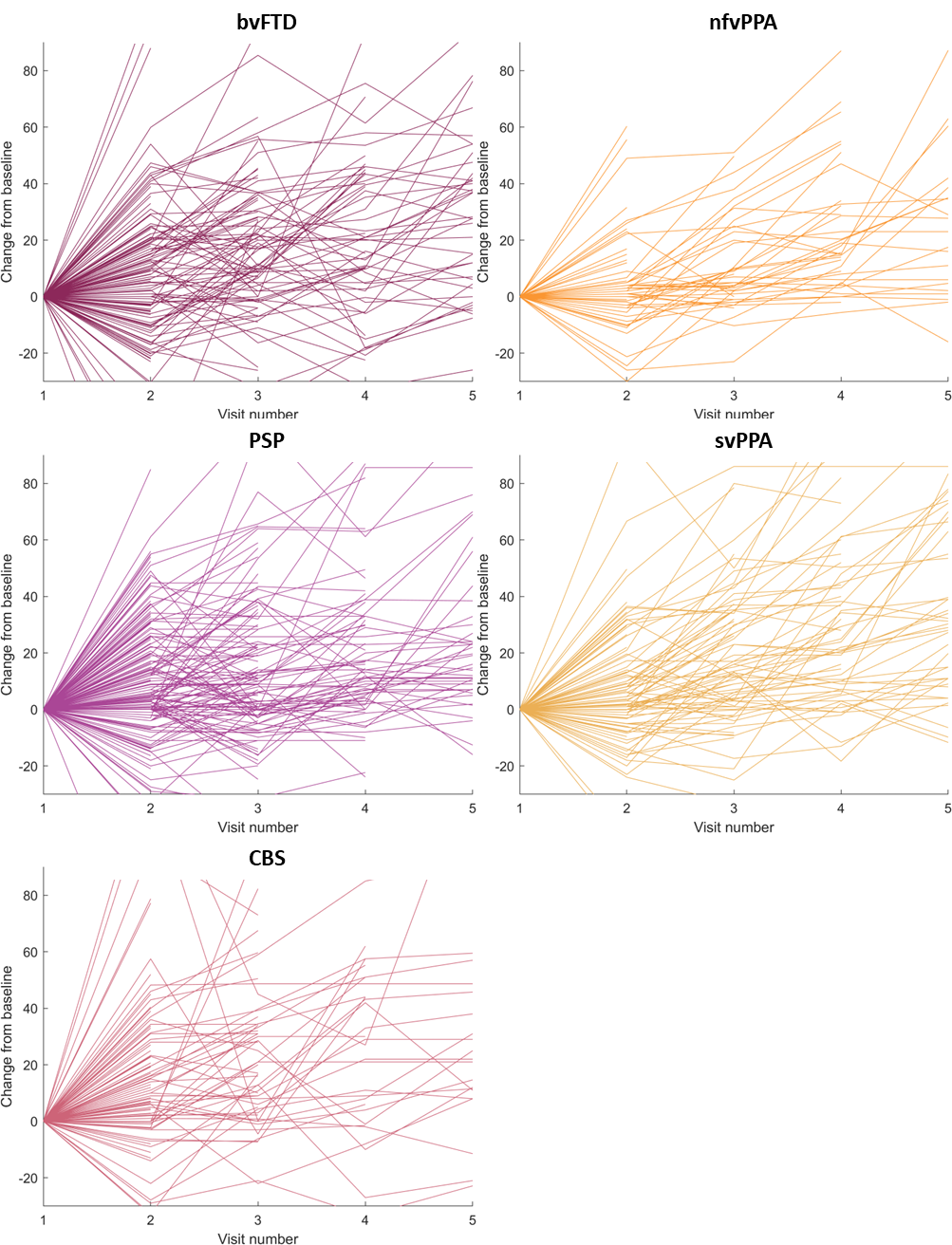
**
